# Chromosome-level genome of the poultry shaft louse *Menopon gallinae* provides insight into the host-switching and adaptive evolution of parasitic lice

**DOI:** 10.1093/gigascience/giae004

**Published:** 2024-02-19

**Authors:** Ye Xu, Ling Ma, Shanlin Liu, Yanxin Liang, Qiaoqiao Liu, Zhixin He, Li Tian, Yuange Duan, Wanzhi Cai, Hu Li, Fan Song

**Affiliations:** Department of Entomology and MOA Key Lab of Pest Monitoring and Green Management, College of Plant Protection, China Agricultural University, Beijing 100193, China; Department of Entomology and MOA Key Lab of Pest Monitoring and Green Management, College of Plant Protection, China Agricultural University, Beijing 100193, China; Department of Entomology and MOA Key Lab of Pest Monitoring and Green Management, College of Plant Protection, China Agricultural University, Beijing 100193, China; Department of Entomology and MOA Key Lab of Pest Monitoring and Green Management, College of Plant Protection, China Agricultural University, Beijing 100193, China; Department of Entomology and MOA Key Lab of Pest Monitoring and Green Management, College of Plant Protection, China Agricultural University, Beijing 100193, China; Department of Entomology and MOA Key Lab of Pest Monitoring and Green Management, College of Plant Protection, China Agricultural University, Beijing 100193, China; Department of Entomology and MOA Key Lab of Pest Monitoring and Green Management, College of Plant Protection, China Agricultural University, Beijing 100193, China; Department of Entomology and MOA Key Lab of Pest Monitoring and Green Management, College of Plant Protection, China Agricultural University, Beijing 100193, China; Department of Entomology and MOA Key Lab of Pest Monitoring and Green Management, College of Plant Protection, China Agricultural University, Beijing 100193, China; Department of Entomology and MOA Key Lab of Pest Monitoring and Green Management, College of Plant Protection, China Agricultural University, Beijing 100193, China; Department of Entomology and MOA Key Lab of Pest Monitoring and Green Management, College of Plant Protection, China Agricultural University, Beijing 100193, China

**Keywords:** *Menopon gallinae*, genome, comparative genomics, host-switching, parasitic lice

## Abstract

**Background:**

Lice (Psocodea: Phthiraptera) are one important group of parasites that infects birds and mammals. It is believed that the ancestor of parasitic lice originated on the ancient avian host, and ancient mammals acquired these parasites via host-switching from birds. Here we present the first chromosome-level genome of *Menopon gallinae* in Amblycera (earliest diverging lineage of parasitic lice). We explore the transition of louse host-switching from birds to mammals at the genomic level by identifying numerous idiosyncratic genomic variations.

**Results:**

The assembled genome is 155 Mb in length, with a contig N50 of 27.42 Mb. Hi-C scaffolding assigned 97% of the bases to 5 chromosomes. The genome of *M. gallinae* retains a basal insect repertoire of 11,950 protein-coding genes. By comparing the genomes of lice to those of multiple representative insects in other orders, we discovered that gene families of digestion, detoxification, and immunity-related are generally conserved between bird lice and mammal lice, while mammal lice have undergone a significant reduction in genes related to chemosensory systems and temperature. This suggests that mammal lice have lost some of these genes through the adaption to environment and temperatures after host-switching. Furthermore, 7 genes related to hematophagy were positively selected in mammal lice, suggesting their involvement in the hematophagous behavior.

**Conclusions:**

Our high-quality genome of *M. gallinae* provides a valuable resource for comparative genomic research in Phthiraptera and facilitates further studies on adaptive evolution of host-switching within parasitic lice.

## Introduction

Lice (Insecta: Phthiraptera) are parasites that infest birds and mammals with more than 4,500 species of chewing lice (Amblycera, Ischnocera, Trichodectera, and Rhynchophthirina) and 500 species of blood-feeding sucking lice (Anoplura) [1, 2]. Chewing lice feed on the feathers, sebaceous secretions, and skin of their avian and mammalian hosts [1], while sucking lice, which parasitize only mammals, have piercing-sucking mouthparts and feed exclusively on blood [3]. These parasites entirely rely on the body of the host, and they affix their eggs to hairs or feathers of the host [1, 3].

As an obligate parasite of domestic chickens (*Gallus gallus*), the poultry shaft louse *Menopon gallinae* (NCBI:txid328185) is a main vector for chicken diseases. These lice live on the skin, penetrate within the skin, or even burrow into the air sacs or under the feathers of chickens. Infestation by these lice can lead to annoyance, decreased weight gain, reduced egg production, egg abandonment in brooding hens, and chick mortality [4]. Additionally, they can cause high morbidity, which adversely affects the economic production of poultry [5].

To date, only 3 louse genomes have been published. Due to technical limitations, the reference genomes of human body louse *Pediculus humanus* (Anoplura) and pigeon wing louse *Columbicola columbae* (Ischnocera) were generated from hundreds or thousands of pooled individuals [6, 7]. In contrast, Sweet et al. [8] recently published a genome assembly from a single individual of feather louse *Brueelia nebulosa* (Ischnocera). Despite different strategies used for genome sequencing and assembly, all 3 louse genomes had high completeness, indicating the contribution of the robust and well-established bioinformatic pipelines in facilitating the genome assembly. Compared with other insects, lice have a reduced number of protein-coding genes (PCGs), including fewer opsin genes, odorant receptors, and detoxification pathways [6, 7]. Our understanding of the genomic signatures of parasitism in Phthiraptera is limited largely to these 3 species. The chromosome-level genome data of the chewing lice suborder Amblycera, which represents the earliest diverging group of lice, are still lacking. Notably, recent studies performed whole-genome sequencing of several lice species and obtained high-quality data, but these data are not assembled and annotated [9] and are not included in our analyses. In addition, a recent study presents a high-quality genome assembly of booklice *Liposcelis brunnea*, a phylogenetic sister group of the 2 parasitic lice. The *Liposcelis brunnea* genome is crucial in understanding the origins and evolution of parasitic lice [10]. Recent studies suggested that parasitic lice have an avian ancestral host and the ancestor of Afrotheria mammals acquired these parasites via host-switching [9, 11]. After this host-switching from birds to mammals, parasitic lice have colonized other lineages of mammals through host-switching and co-diversified with their host. Parasitic lice have specific morphological and behavioral adaptations for attachment and avoiding host defenses [1, 2]. In contrast to bird lice, mammal lice are morphologically adapted to live on their mammal hosts with tibial tarsal claws to attach to host hairs and highly derived mouthparts for feeding directly from host blood vessels [12].

During host-switching of lice, their genomes accumulate mutations, some of which may be directly linked to functional adaptations. Identifying such genomic feature and linking them to phenotypic differences is critical for deciphering the genomic drivers of species adaptability. Expansion or contraction of key gene families may facilitate the emergence of novel functions, leading to successful host-switching of lice. Therefore, it is necessary to reveal significant variations in the genome during the host-switching of parasitic lice from birds to mammals.

In this study, we presented a high-quality chromosome-level genome of *M. gallinae* (representing the earliest diverged lice Amblycera) using a combination of Illumina short-read sequencing, PacBio high-fidelity (HiFi) long-read sequencing, and Hi-C technology. Combining the genome of human body louse *P. humanus*, the latest diverged lice (Anoplura), together with other various representative insect species, we performed comparative genomic analyses to evaluate the evolution of genes putatively involved in host-switching of parasitic lice from birds to mammals. These data would supply a useful genetic resource for future research of parasitic lice.

## Methods

### Samples collection and identification

For genome sequencing of the poultry shaft louse *M. gallinae*, approximately 1,600 individuals were collected from natural populations infesting chickens (*G. gallus*) in Chongqing, China. The 1,600 individuals were simultaneously collected from the same chicken farm. Species identification was determined using a combination of morphological identification under the microscope according to Price et al. [1] and molecular identification through sequencing of COI fragments (∼550 bp).

### DNA extraction, RNA extraction, library construction, and sequencing

Total genomic DNA was extracted from individual louse using the TIANamp Genomic DNA Kit (Tiangen) following the manufacturer’s instructions. The COI were amplified by PCR, and the PCR products were sequenced by Sanger sequencing at Tsingke Biotechnology.

Genomic DNA used for the SMRTbell library preparation was extracted from around 1,000 adults with the Blood & Cell Culture DNA Midi Kit (Qiagen). After assessing the quality of the isolated DNA, a ∼20-kb library was constructed using the SMRTbell Express Template Prep Kit 2.0 (Pacific BioSciences). HiFi long clean reads produced by circular consensus sequencing on the PacBio Sequel II platform were used for contig-level genome assembly.

For genome survey, genomic DNA was extracted from 50 adults and an Illumina sequencing library was constructed according to the manufacturer’s instructions (Illumina). The library was then sequenced on the Illumina NovaSeq 6000 platform in paired-end 150-bp mode to generate approximately 50 Gb data.

For genome annotation, total RNA was extracted from 50 adults using the Tiangen RNA extraction kit. After reverse transcription of mRNA into cDNA, another Illumina RNA sequencing (RNA-seq) library was constructed and sequenced with the same parameters, generating approximately 6 Gb of data. In addition, a PacBio Iso-Seq library was constructed using the SMRTbell Express Template Prep Kit 2.0 (Pacific BioSciences) from 50 adults and sequenced on the PacBio Sequel II platform (RRID:SCR_017990), generating approximately 60 Gb of data.

To construct a chromosomal-level assembly of the genome, we constructed the Hi-C library. In brief, 600 adults of *M. gallinae* were immersed in 2% formaldehyde for cross­linking of cellular protein. The purified nuclei were digested with 100 units of DpnII enzyme. Then Hi-C samples were extracted by biotin labeling, flat-end ligation, DNA purification, and random shearing of DNA into 300- to 600-bp fragments. Finally, the Hi-C libraries were quantified and sequenced using the Illumina NovaSeq platform (RRID:SCR_016387) with paired-end 150-bp reads.

### Genome assembly and evaluation

The Illumina reads were used for the genome survey, aiming to estimate the genome size, heterozygosity, and duplication using the *k*-mer method. Specifically, 17-base oligonucleotide *k*-mers were counted using JELLYFISH (RRID:SCR_005491) version 2.1.3 [13]. The genome features were then evaluated using GenomeScope (RRID:SCR_017014) version 2.0 [14]. We used several approaches to assemble the *M. gallinae* genome. The following tools were tried: WTDBG2 (RRID:SCR_017225) version 2.5 [15], HiCanu version 2.1.1 [16], Hifiasm (RRID:SCR_021069) version 0.13 [17], and Flye (RRID:SCR_017016) version 2.9.2 [18]. The purge_dups (RRID:SCR_021173) version 1.2.6 [19] was used to remove potential haplotypic duplications and contig overlaps. The statistic details resulting from different tools are summarized in Table 1. WTDBG2 and Flye produced a remarkably larger contig N50 (27.42 Mb and 27.21 Mb) compared with the other 2 tools (6.34 Mb and 1.25 Mb). Then, among WTDBG2 and Flye, WTDBG2 produced a larger genome size (155 Mb) and therefore the genome assembly from WTDBG2 was used. Clean reads sequenced from the Hi-C library were aligned to the contig-level genome with an end-to-end algorithm implemented in BWA-MEM version 0.7.17 [20]. Juicer (RRID:SCR_017226) version 1.6 [21] and 3D-DNA version 180419 [22] were used to assemble the scaffolds into a chromosome-level genome. The chromosome-level genome was reviewed using Juicebox (RRID:SCR_021172) version 1.11.08. The completeness of the genome was assessed using BUSCO version 3.0.2 with the insecta_odb10 database [23].

### Repeat sequences annotation

We used RepeatMasker (RRID:SCR_012954) version 4.0.7 [24] and RepeatProteinMasker version 4.0.7 [24] to identify and annotate repeat sequences based on RepBase (RRID:SCR_021169) edition 2017012732 [25]. RepeatModeler (RRID:SCR_015027) version 2.0.4 [26] was used to construct a *de novo* repeat library. LTR FINDER (RRID:SCR_015247) version 1.0726 [27] and LTR retriever version 2.9.028 [28] were used to identify long terminal repeat (LTR) retrotransposons. Tandem Repeats Finder (RRID:SCR_022065) version 4.09.1 [29] was used to annotate tandem repeats.

### Protein-coding gene annotation

We used 3 kinds of evidence to annotate PCGs, including *ab initio*, RNA-seq–based, and homolog-based methods. For RNA-seq–based gene prediction, we mapped short reads of *M. gallinae* from Illumina transcriptome sequencing to the genome using HISAT version 2.2.1 [30]. The mapped reads were used to assemble transcripts with StringTie (RRID:SCR_016323) version 2.4.0 [31]. The IsoSeq data were also processed using IsoSeq3 (RRID:SCR_022749) version 3.8.2 with certain parameters like filtering, clustering, and polishing. The 2 transcripts were chosen as mRNA evidence. For the homolog-based approach, we downloaded protein sequences of 10 species (*Drosophila melanogaster* [32], *P. humanus* [6], *C. columbae* [7], *Bombyx mori* [33], *Acyrthosiphon pisum* [34], *Tribolium castaneum* [35], *Anopheles gambiae* [36], *Acromyrmex echinatior* [37], *Apis mellifera* [38], and *Nasonia vitripennis* [39]) from NCBI and InsectBase 2.0 [40]. For the *ab initio* method, we used Exonerate version 2.4.0 [41] to align homologous proteins and transcripts. Additionally, we utilized the bam2hints program in AUGUSTUS (RRID:SCR_008417) version 3.2.3 [42] to transfer the sorted and mapped bam file of RNA-seq data into a hints file. These trained gene sets and hint files were then combined as inputs for AUGUSTUS version 3.2.3 [42] to predict coding genes from the assembled genome. Finally, the high-confidence gene set was generated by merging *ab initio*, RNA-seq–based, and homology-based genes using MAKER (RRID:SCR_005309) version 2.31.10 [43].

### Identification of orthologous genes and inference of phylogenetic relationships

To infer the phylogenetic relationships of *M. gallinae* and other insects, we selected an additional 9 species for phylogenetic analysis. We utilized all the protein sequences of 10 insects and selected 2 dipteran insects as an outgroup. OrthoFinder (RRID:SCR_017118) version 2.5.4 [44] was used to find gene families including single-copy genes and paralogous gene families. Based on the results of OrthoFinder, gene family clusters were divided into 4 categories: (i) single-copy genes in all species, (ii) multiple-copy genes in at least 1 species, (iii) species-specific genes (genes absent in other N–1 species), and (iv) other genes.

The phylogenetic tree was inferred using single-copy orthologues in each species. Sequence alignment was performed using MAFFT (RRID:SCR_011811) version 7.520 [45], and the resulting alignment was trimmed with the option “automated1” using trimAl version 1.4.rev15 [46]. We estimated the phylogenetic tree using the concatenated sequences of aligned proteins in IQ-TREE (RRID:SCR_017254) version 2.1.4 [47] with options “-m TEST -bb 1000 -alrt 1000.” The best-fit model (Q.insect+I+G4) was compared and selected according to the Bayesian information criterion by using ModelFinder [48]. The divergence time was estimated using MCMCTree (clock = 3, RootAge = 4.0, rgene_gamma = 1 15.83709, sigma2_gamma = 1 4.5) from PAML version 4.9 [49] with the approximate likelihood method. Two calibration times based on previous studies [50–52] from the TIMETREE database (RRID:SCR_021162) were utilized for estimation: *A. pisum*–*P. humanus* (172.6–416.6 Mya) and *A. pisum–Apolygus lucorum* (112.5–391.7 Mya).

### Gene family expansion, contraction, and annotation

CAFÉ (RRID:SCR_005983) version 4.2.1 [53] was employed to examine gene family expansion and contraction among species with the results from OrthoFinder and the phylogenetic tree with divergence times as inputs. Phylogenetic tree topology and branch lengths were considered when inferring the significance of changes to gene family size in each branch. Families with conditional *P* values lower than 0.05 were considered to have had a significantly accelerated rate of expansion or contraction. The results figure was analyzed using the R package GOplot (RRID:SCR_024419) version 1.0.2 [54].

For gene family annotation, we downloaded the protein sequences of corresponding gene families in well-annotated insect species *P. humanus, D. melanogaster*, and *A. pisum* from the NCBI database. We searched these gene families against the protein sequences of 10 species with BLAST version 2.12.0 (evalue 1e−5) [55]. Notably, for the proteins annotated in the Pfam database (RRID:SCR_004726), we confirmed the domains of this protein by HMMER (RRID:SCR_005305) version 3.0 [56]. The detected proteins in a species were regarded as presence and the undetected proteins were regarded as absence. For the putative lost genes in species, we used Exonerate (RRID:SCR_016088) version 2.4.0 [41] to search those protein sequences in other insects against the genome. If the sequence was not mapped to the genome, this might be a gene loss. If the target protein sequence was not mapped to the genome or aligned to the genome but the genome sequence does not have a complete gene structure (which indicates a pseudogene), then this protein is considered to be lost in this species. Finally, each candidate gene was manually inspected and divided into subfamilies. In summary, hematophagy-related genes included iron/heme binding, transport and metabolism, oxidative stress, urea cycle enzymes, and other genes. Chemosensory gene families included gustatory receptors (GRs), odorant-binding proteins (OBPs), chemosensory proteins (CSPs), odorant receptors (ORs), ionotropic receptors (IRs), and sensory neuron membrane proteins (SNMPs). Detoxification gene families included cytochrome P450 monooxygenases (P450s), glutathione-S-transferases, esterases, UDP-glycosyltransferases, and ATP-binding cassette transporter (ABC transporter). Furthermore, we identified the heat shock protein (Hsp), 5 major digestive enzymes, and 36 immunity-related genes. Protein sequences of the annotated P450s, ABC transporter, Hsp, OR, and GR genes were aligned and trimmed using MAFFT (RRID:SCR_011811) version 7.520 [45] and trimAl (RRID:SCR_017334) version 1.4.rev15 [46] with default parameters. Phylogenetic trees were constructed using IQ-TREE version 2.1.4 [47] with options “-m TEST -bb 1000 -alrt 1000” and visualized using the R package GGTREE (RRID:SCR_018560) version 3.3.1 [57].

### Positive selection and *dN/dS* ratio analysis

We used MAFFT version 7.520 [45] to align the protein sequences and subsequently converted the multiple protein sequence alignment and corresponding coding sequences (CDS) into a codon alignment using Pal2Nal version 14 [58]. To calculate *dN/dS* ratios across pairwise alignments of each gene pair between bird lice *M. gallinae* and mammal lice *P. humanus*, we employed the Yn00 algorithm in PAML version 4.9 [49]. To identify potential positively selected genes in *M. gallinae* and *P. humanus*, we utilized the branch-site model of CodeML in PAML (RRID:SCR_014932) version 4.9 [49] with single-copy orthologues of 10 insect species. Specifically, we set *M. gallinae*/*P. humanus* as the foreground branch and the remaining species as background branches. We chose *P* < 0.05 as the significance threshold after FDR correction to identify a particular orthogroup as positively selected.

## Results

### Genome sequencing and assembly

**Figure 1:**
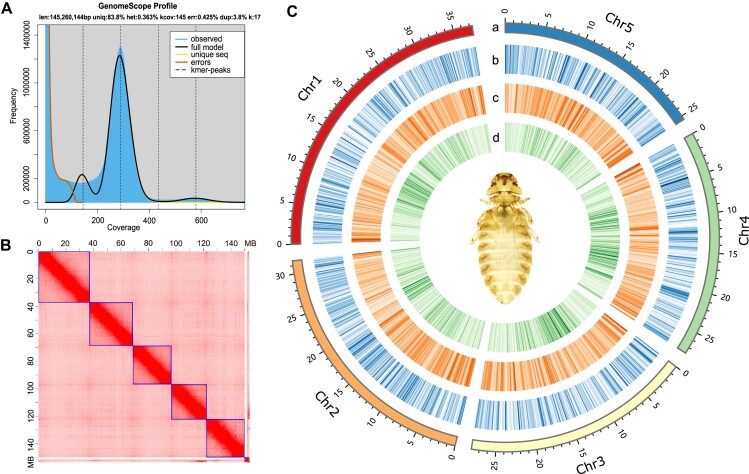
Genome description of *M. gallinae*. (A) GenomeScope estimation of genome size and heterogeneity using a *k*-mer of 17. (B) Hi-C interaction map produced by 3D-DNA. (C) Circular representation of the chromosomes. Tracks A–D represent the distribution of chromosome karyotypes, gene density, GC density, and repeat sequences density, respectively. Densities were calculated in 100-kb windows.

We assembled a high-quality chromosome-level genome of *M. gallinae* by using a combination of PacBio long reads (28.51 Gb, 184-fold), Illumina short reads (50.79 Gb, 328.32-fold), and Hi-C reads (20.86 Gb, 134-fold). The genome size was estimated to be 145 Mb with a heterozygosity rate of 0.363% by calculating the frequency with 17 *k*-mer analysis (Fig. 1A).

At the contig level, we generated a final genome assembly of 155 Mb using WTDBG2 as this tool produced the largest contig N50 compared with other tools we tried (see Methods). The genome consists of 100 contigs with an N50 of 27.42 Mb (Table 2). This final genome size of *M. gallinae* (155 Mb) is comparable to our preliminary estimation (145 Mb). However, it is larger than the previously published genome size of mammal lice *P. humanus* (108 Mb) [6] and feather louse *B. nebuosa* (114 Mb) [8], as well as smaller than that of the pigeon wing louse *C. columbae* (208 Mb) [7] and the booklice *L. brunnea* (174 Mb) [10] genome (Table 2). Our *M. gallinae* genome possessed a remarkably higher contig N50 (27.42 Mb) compared to other published genomes of parasitic lice and booklice, including *P. humanus* (34 kb), *C. columbae* (511 kb), *B. nebuosa* (293 kb), and *L. brunnea* (1.78 Mb) (Table 2). The GC content in *M. gallinae* (41%) was slightly higher than that in *B. nebuosa* (38%), *C. columbae* (36%), and booklice *L. brunnea* (35%) and remarkably higher than that in *P. humanus* (28%), which possesses an extremely AT-rich genome [6] (Table 2). The GC content of the parasitic lice genome was highly variable. Five complete chromosomes were obtained in our assembly (N50 = 27.95 Mb), consisting of 97% of the whole genome. The number of chromosomes is comparable to *P. humanus* (6), smaller than booklice *L. brunnea* (9), and notably smaller than *C. columbae* (12) [6, 7, 10] (Table 2). Chromosome lengths range from 25.47 to 37.36 Mb (Fig. 1B, C). Moreover, BUSCO evaluation showed high completeness and accuracy of the genome assembly, with 97.2% of genes being successfully identified, including 96.8% of single-copy genes and 0.4% of duplicated genes. These results justify a high-quality *M. gallinae* genome that could be used in the downstream analyses.

**Table 1:** Statistics for the assembly of *M. gallinae* using PacBio data.

Feature	WTDBG2	Hifiasm	HiCanu	Flye
Genome size	155 Mb	217 Mb	254 Mb	151 Mb
Number of contigs	100	805	424	24
Contig N50	27.42 Mb	6.34 Mb	1.25 Mb	27.21 Mb
BUSCO	97.2%	97.5%	98.1%	98.0%
GC content	41%	41%	41%	41%

**Table 2:** Genome features of 4 parasitic lice and 1 booklice.

Feature	*Pediculus humanus*	*Menopon gallinae*	*Columbicola columbae*	*Brueelia nebuosa*	*Liposcelis brunnea*
Assembly level	Scaffold	Chromosome	Chromosome	Scaffold	Chromosome
Heterozygosity	-	0.363%	—	1.2%	0.268%
Survey	103–109 Mb	145 Mb	230 Mb	100 Mb	172 Mb
Genome size	108 Mb	155 Mb	208 Mb	114 Mb	174 Mb
Contig N50	34 kb	27.42 Mb	511 kb	293 kb	1.78 Mb
Scaffold N50	497 kb	27.95 Mb	17.67 Mb	637 kb	19.7 Mb
Chromosomes	6	5	12	—	9
BUSCO	95.9%	97.2%	96.4%	96.1%	97.2%
GC content	28%	41%	36%	38%	35%
Protein-coding genes	10,773	11,950	13,362	10,938	15,543
Repetitive elements	7.3%	4.1%	9.7%	15.1%	15.9%

### Genome annotation

To predict bona fide protein-coding genes in the *M. gallinae* genome, we employed 3 different approaches: *de novo* prediction, homologous gene prediction, and RNA-seq–based prediction (see Methods for details). In total, we predicted 11,950 PCGs that were supported by 3 approaches. The number of PCGs in *M. gallinae* was comparable to that observed in mammal lice *P. humanus* (10,773 PCGs) [6] and feather louse *B. nebuosa* (10,938 PCGs) [8] but lower than the number reported in the genome of the pigeon wing louse *C. columbae* (13,362 PCGs) [7] and the booklice *L. brunnea* (15,543 PCGs) [10] (Table 2). This result indicated that the coding genes in 2 parasitic lice might have experienced an ancestral loss due to their limited habitats and simple dietary regimes compared to the widespread booklice *L. brunnea*.

Annotation of the 11,950 PCGs in *M. gallinae* revealed an average of 8.26 exons and 7.26 introns per gene. The average length of the mRNA transcripts was 2,763.21 bp, while the average length of the CDS was 1,815.98 bp (Supplementary Table S1). Functional annotation revealed that 10,664 (89.24%), 9,178 (76.80%), and 8,999 (75.31%) genes matched with proteins recorded in databases NR, SwissProt, and Pfam, respectively. Furthermore, 4,166 (34.86%) and 6,562 (54.91%) genes were successfully annotated by Gene Ontology (GO) terms and KEGG pathways, respectively (Supplementary Table S2).

Repeat sequences (transposable elements, TEs) only made up 4.1% of the *M. gallinae* genome. This fraction of repeats is considerably lower than that observed in other published genomes of parasitic lice and booklice, such as *P. humanus* (7.3%) and *C. columbae* (9.7%), and particularly *B. nebuosa* (15.1%) and *L. brunnea* (15.9%) (Table 2) [6–8, 10]. For specific types of TEs, 0.01% of the *M. gallinae* genome includes short interspersed nuclear elements, 0.27% includes long interspersed nuclear elements, 0.58% includes LTRs, 0.56% is DNA transposon, and the other 2.30% includes tandem repeats (TRs) (Supplementary Table S3). TRs are much more abundant than any other types of TEs in *M. gallinae*.

### Orthologue identification and phylogenetic inference

**Figure 2:**
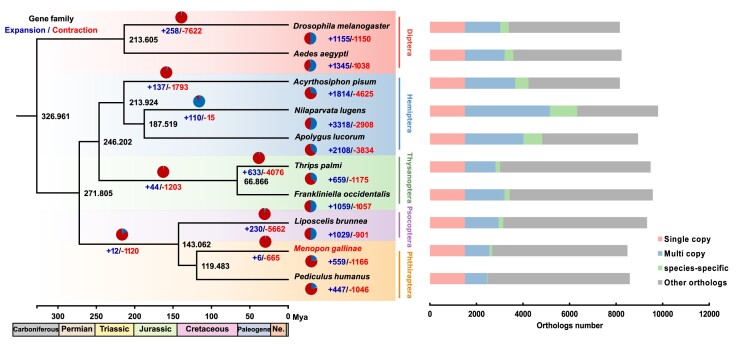
Phylogenetic tree with the dynamic evolution of gene families among *M. gallinae, P. humanus*, and other species. In the left panel, blue and red numbers on the branch show the number of expanded and contracted gene families for each clade. Pie charts beside or on each branch of the tree show the proportion of expanded (blue) and contracted (red) gene families. The black numbers are divergence times. In the right panel, the numbers of gene families (orthogroups) are shown as barplots. Orthogroups of different categories are in different colors.

To get a landscape of the evolutionary gains and losses of functional genes in 2 parasitic lice and understand how these evolutionary dynamics are related to the phenotypic innovations of the species, we looked for orthologous genes in *M. gallinae* (representing the earliest diverged lice Amblycera, bird lice), *P. humanus* (representing the latest diverged lice Anoplura, mammal lice), and other 8 representative insect species. The gene family clusters were divided into 4 categories, single-copy genes, multiple-copy genes, species-specific genes (unique genes), and other genes. A phylogenetic tree generated using single-copy orthologous genes showed that all species of Paraneoptera (Psocodea, Hemiptera, and Thysanoptera) formed a clade. A total of 137,947 genes from all 10 species were clustered into 16,518 unique gene families (orthogroups) using OrthoFinder (see Methods). Particularly, there were 115 orthogroups specific to bird lice, while only 23 orthogroups were specific to mammal lice (Fig. 2; Supplementary Table S4).

### Expansion, contraction, and positively selected of gene families

**Figure 3:**
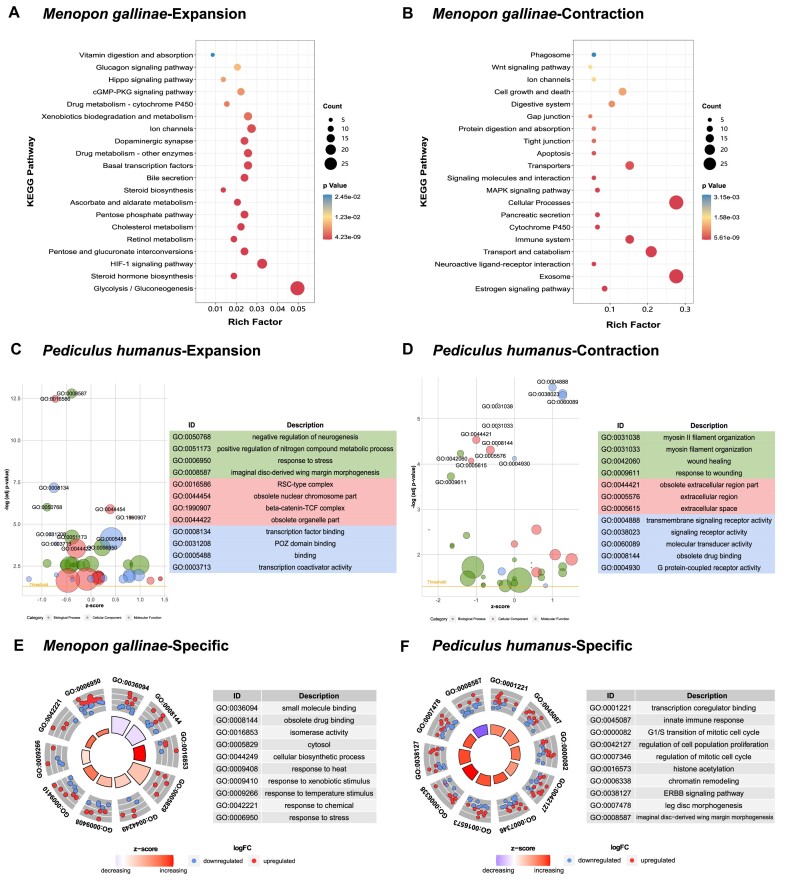
Enrichment analysis of gene families of different categories. KEGG pathway of expanded (A) and contracted (B) gene families of *M. gallinae*. GO enrichment of expanded (C) and contracted (D) gene families of *P. humanus*. GO enrichment of specific gene families of *M. gallinae* (E) and *P. humanus* (F).

We used CAFÉ version 4.2.1 [53] to study the expansions and contractions of gene families during the evolution of parasitic lice. Compared to the common ancestor of the parasitic lice, we found 559 expanded and 1,166 contracted gene families in bird lice and 447 expanded and 1,046 contracted gene families in mammal lice. Similarly, 1,029 expanded and 901 contracted gene families were founded in booklice compared to the ancestral node of booklice and parasitic lice (Fig. 2). Enrichment analysis of GO and KEGG revealed that expanded gene families in bird lice are enriched in obsolete drug binding, drug metabolism—other enzymes, and Hippo signaling pathway (Fig. 3A; Supplementary Tables S5 and S6), while contracted gene families are enriched in chemosensory behavior, cytochrome P450, digestive system, and immune system (Fig. 3B; Supplementary Tables S7 and S8). Correspondingly, for mammal lice, while the expanded gene families are enriched in negative regulation of neurogenesis and positive regulation of nitrogen compound metabolic process (Fig. 3C; Supplementary Table S9), those contracted gene families are enriched in G protein–coupled receptor activity, obsolete drug binding, and signaling receptor activity (Fig. 3D; Supplementary Table S10). The fact is that the contracted gene families of chemosensory-related pathways in 2 parasitic lice imply that these genomic features may be relevant to their parasite behavior after the split from their common ancestor. Accordingly, we estimated the expanded and contracted gene families in the ancestor of 2 parasitic lice and found that the contracted genes are enriched in response to xenobiotic stimulus, response to insecticide, defense response, cytochrome P450, and digestive system (Supplementary Tables S11 and  S12). Next, our analysis on booklice revealed that the expanded gene families are enriched in response to xenobiotic stimulus, lectins, and drug metabolism–cytochrome P450 (Supplementary Tables S13 and S14).

To understand whether the species-specific genes among the 3 lice contribute to their distinct behavior, we looked for the gene families present in 1 lice species but absent in all other 9 insect species we used. We uncovered 115, 23, and 198 species-specific gene families in bird lice, mammal lice, and booklice, respectively (Supplementary Table S4). Gene enrichment analysis showed that bird lice–specific gene families are enriched in pathways of response to temperature stimulus, response to heat, and response to xenobiotic stimulus (Fig. 3E; Supplementary Tables S15 and S16). Mammal lice–specific gene families are enriched in innate immune response and epidermal growth factor receptor signaling pathway (Fig. 3F; Supplementary Table S17). Booklice-specific gene families are enriched in cellular response to oxygen-containing compound, digestive system, and cytochrome P450 (Supplementary Tables S18 and S19). This result agrees with a previous finding that P450 genes were expanded in booklice [10].

Positive selection is an important source of evolutionary innovation and one of the major forces driving species divergence. We identify the positively selected genes in single-copy genes from these species for branch-site model analysis by maximum likelihood (PAML) (see Methods). We found that 135 and 201 genes were positively selected in bird and mammal lice, respectively (likelihood ratio test, *P* < 0.05). Seven hematophagy-related genes in mammal lice were positively selected, such as heme-related genes (coproporphyrinogen III oxidase), iron-related genes (nuclear hormone receptor, NADH ubiquinone 75 kD subunit, peroxinectin like, mitoferrin, transferrin 2) and salivary IP5P (Supplementary Table S20), which may be due to the unique hematophagous behavior of mammal lice. Based on the above results of gene expansion/contraction and positively selected genes, we will focus on hematophagy, digestion, chemosensory system, temperature, immunity, and detoxification gene families in the following analyses.

### Hematophagy-related genes

**Figure 4:**
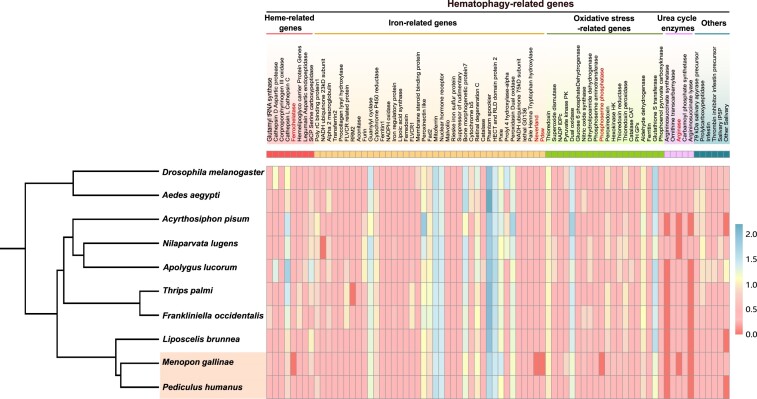
Distribution of hematophagy-related genes in the genomes of *M. gallinae, P. humanus*, and other species. The heatmap shows the numbers of hematophagy-related genes. The numbers were transformed with log_10_(n + 1).

We compared hematophagy-related genes to observe the signature change of feeding habit during host-switching. We found that proteins involved in iron/heme binding, transport and metabolism, oxidative stress, urea cycle enzymes, and other hematophagy-related genes are generally conserved in 2 lice genomes (Fig. 4; Supplementary Table S21). Among the hematophagy-related genes in mammal lice, 5 were absent in bird lice: heme-related genes (ferrochelatase), iron-related genes (neverland, ND-pdsw), oxidative stress–related genes (phosphoserine phosphatase), and urea cycle enzymes (arginase). Ferrochelatase is a crucial enzyme involved in heme synthesis in insects [59]. ND-pdsw plays a key role in oxidative phosphorylation and is overrepresented in blood-feeding insects [60].

### Major digestive enzyme analysis

As important as hematophagy-related genes, digestive enzyme are also essential for diet of lice. The digestive enzyme of parasitic arthropods is mainly involved in the digestion of host blood and other ingested proteins present in the skin [61–63]. Feather feeding in bird lice and hematophagy in mammal lice may result in different adaptations and alterations in the composition of digestive enzymes in 2 lice. We found bird lice (168 genes) have more digestive enzymes than mammal lice (152 genes) (Supplementary Fig. S1; Supplementary Table S21). Compared to other insects or even the booklice (236 genes), digestive enzymes of 2 parasitic lice were substantially contracted (Supplementary Fig. S1).

### Genes associated with chemosensory systems

**Figure 5:**
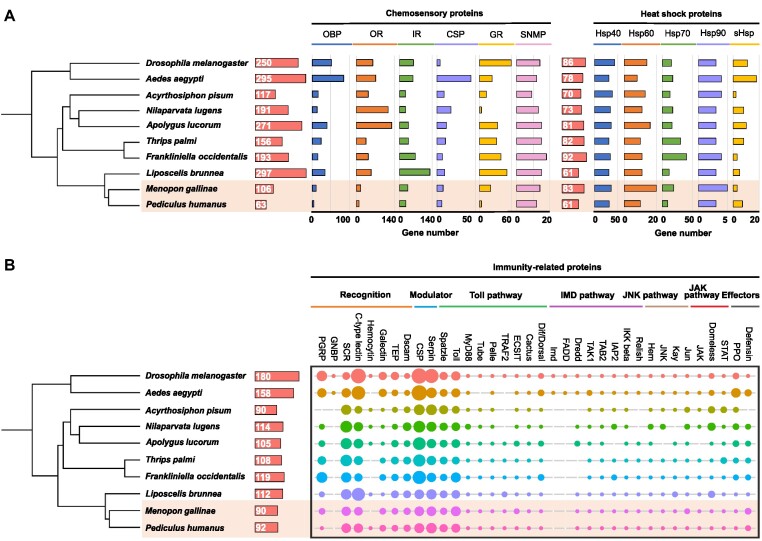
Distribution of (A) chemosensory proteins and heat shock proteins and (B) immunity-related proteins in *M. gallinae, P. humanus*, and other species.

Genes involved in chemosensory systems, especially the OR, GR, and IR subfamilies, play a critical role in feeding, mating, and predator avoidance of insects [64, 65]. We collected 6 subfamilies of chemosensory-related genes and the 2 parasitic lice have remarkably reduced chemosensory-related genes. Mammal lice have fewer chemosensory-related genes (totally 63 genes) than bird lice (106 genes) (Fig. 5A; Supplementary Table S21). Bird lice have 21 GR, 16 OR, and 36 IR genes, while mammal lice have only 4 GR, 9 OR, and 27 IR genes (Fig. 5A; Supplementary Figs. S2A and S2B). Notably, GR genes showed the most reduction in mammal lice. GR genes primarily mediate gustation, specifically detecting sweet and bitter tastants, as well as to sense carbon dioxide (CO_2_) [66–69]. A previous study has reported that some GR genes (such as sugar receptor genes and CO_2_ receptor genes) were absent in mammal lice [6]. Here we confirmed that the bird lice genome encodes 2 sugar receptors and 2 CO_2_ receptors. In addition, we identified a fructose receptor gene in the genome of bird lice that is absent from the protein and genome sequences of mammal lice (Supplementary Fig. S2A). The remaining members of the GR subfamily all belong to GR28 genes: bird lice have 16 GR28 genes while mammal lice only have 4 GR28 genes. GR28 are temperature sensors that can help identify hosts dependent on warmth, the strategy of which has been widely used by parasites such as tsetse flies and mosquitoes [70].

### Temperature-related genes

**Figure 6:**
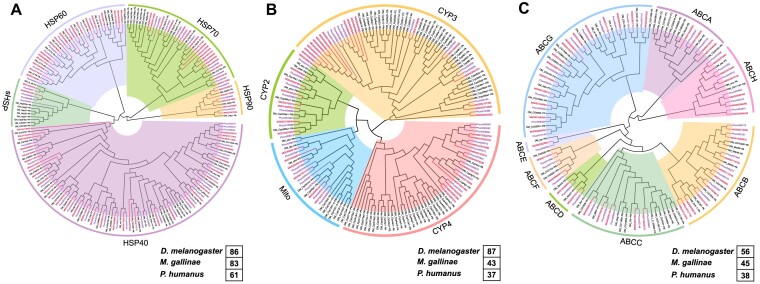
Phylogenetic relationships of *M. gallinae* (MG) (A) heat shock protein (HSP), (B) cytochrome P450 (P450), and (C) ATP binding cassette (ABC) transporter gene families in comparison with *D. melanogaster* (DM) and *P. humanus* (Phum).

As molecular chaperones, Hsps play important roles in helping insects cope with various ambient stresses, such as extreme temperatures, oxidation, heavy metals, and other abiotic factors [71, 72]. We have collected 5 Hsp subfamilies and found fewer Hsp genes in mammal lice (61 genes) than in bird lice (83 genes). The main difference is that mammal lice have 10 Hsp60 and 9 Hsp70 genes, while bird lice have 20 Hsp60 and 19 Hsp70 genes (Figs. 5A and 6A; Supplementary Table S21). Hsp40 and Hsp90 also show higher copy numbers in bird lice than in mammal lice while only sHsp is more abundant in mammal lice (7 genes) than in bird lice (3 genes) (Fig. 5A; Supplementary Table S21).

### Immunity-related genes

In defense against pathogens, insects rely mainly on their innate immune system [73, 74]. Ninety-two immunity-related genes were identified from the mammal lice genome while 90 were identified from the bird lice genome (Fig. 5B; Supplementary Table S21). All components of the Toll, JAK/STAT, and JNK pathways existed in both lice, but several gene families involved in the humoral immune system were considerably diminished or missing in 2 lice genomes. In case of pathogen recognition-related genes, bird lice have 3 PGRP while mammal lice have only 1, and the GNBP protein was absent in 2 lice. Several components of the Imd pathway (Imd and its adaptor protein FADD) are not found in the protein and genome sequences of bird lice, as reported in mammal lice [75]. A similar result was observed in the pea aphid *A. pisum* and kissing bug *Rhodnius prolixus*, where a more extensive loss of the Imd pathway genes purportedly allowed the development of its obligate endosymbiont [76]. Furthermore, the hemocytin gene was found in the mammal lice genome, which is absent in bird lice.

### Detoxification gene family analysis

Detoxification genes are involved in the metabolic detoxification of xenobiotics, such as plant allelochemicals and synthetic insecticides [77]. The number of detoxification genes was similar in 2 lice as 5 detoxification families (P450s, GSTs, ESTs, UGTs, and ABC) of bird lice included 43, 20, 13, 9, and 45 genes, whereas mammal lice had 37, 18, 11, 4, and 38 genes for each family (Supplementary Fig. S3A; Supplementary Table S22). When mapping 43 P450 genes to the chromosomes of bird lice, 1 gene cluster with 10 CYP3 genes was found on chromosome 4 (Fig. 6B and Supplementary Fig. S3B), consistent with the expansion of 10 CYP3 genes (Supplementary Table S7). This expansion of CYP3 has been associated with pesticide resistance and xenobiotic metabolism, as studied in several dipteran and lepidopteran insects [78, 79]. For ABC transporter, only ABCH subfamilies are slightly more abundant in 2 lice (6 genes) than in *D. melanogaster* (3 genes) (Fig. 6C and Supplementary Fig. S3A; Supplementary Table S22), while the ABCG5 gene was positively selected in mammal lice (Supplementary Table S20).

### *dN/dS* ratios analysis

**Figure 7:**
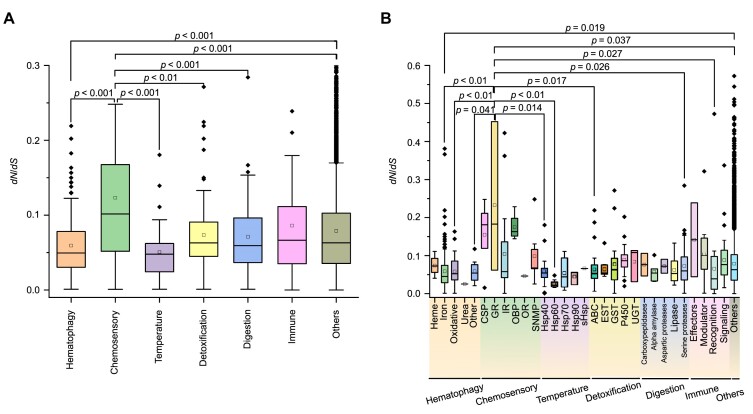
Comparing the *dN*/*dS* ratios between *M. gallinae* and *P. humanus*. (A, B) Gene families and subfamilies related to hematophagy, chemosensory systems, temperature, detoxification, digestive, immunity, and other gene families.

We calculated the *dN/dS* ratios (between mammal and bird lice) of hematophagy, chemosensory systems, detoxification, temperature, digestion, and immunity-related genes with those of other gene families. Higher *dN/dS* represents faster evolution rate. We found that chemosensory genes exhibit slightly higher *dN/dS* ratios than other gene categories (*P* < 0.001, *t*-test) (Fig. 7A), suggesting the rapid evolution of chemosensory genes in lice. Among chemosensory genes, GR genes have higher *dN/dS* ratios than other subfamilies (Fig. 7B), suggesting that even among the fast-evolving chemosensory genes, the GR subfamily is under relaxed selective constraints. The *dN/dS* ratios of hematophagy-related genes are significantly lower than those of chemosensory genes and other genes (*P* < 0.001 in both comparisons, *t*-test) but show no significant difference with the remaining groups (Fig. 7A). Among these hematophagy-related genes, iron-related genes have the lowest *dN/dS* ratios than other genes (Fig. 7B), indicating that conservation is more evident across hematophagy-related genes.

## Discussion

### Successful sample collection and the chromosome-level genome assembly of *M. gallinae*

In this study, we present the first chromosome-level genome of Amblycera (*M. gallinae*) following previous genomic studies on, which were not full genomic assemblies [9]. Genome assembly is typically challenged by high heterozygosity and replication, particularly in small insects that require the extraction of DNA from multiple individuals to construct sequencing libraries [6, 7]. Many lice are difficult to obtain due to their low abundance on a single host (usually <10 individuals) [80]. In this study, we collected a large number of adult *M. gallinae* from a chicken farm in Chongqing and used a long-read sequencing strategy (PacBio HiFi and Hi-C) to assemble its genome. This strategy has been shown to produce high integrity and continuity in genome assembly [81–83], making it suitable for high-quality *de novo* assembly of abundant small parasitic lice genomes. Although our *M. gallinae* individuals are not from an inbred population, they were simultaneously collected from several chickens in the same room of the chicken farm. Therefore, the lice on the hosts are likely to have a highly homogeneous genetic background. Accordingly, the heterozygosity in our samples was as low as 0.363%, the level of which was lower than that of feather louse *B. nebuosa* (1.2%) [8] (only 1 individual was used), melon thrips *Thrips palmi* (1.32%) [84], and mirid predator *Cyrtorhinus lividipennis* (1.7%) [85]. In addition, it is worth noting that the low *dN*/*dS* ratios in both important gene families of *M. gallinae* and *P. humanus* indicate that they have undergone strong purifying selection, meaning that mostf deleterious mutations have been eliminated. While the population of *M. gallinae* used for genome sequencing and assembly was not inbred, it's important to note that the individuals were sourced from a genetically pure origin. The genome size of *M. gallinae* (155 Mb) is intermediate between the genome sizes of other lice species, including *P. humanus* (∼110 Mb) [6], *B. nebuosa* (∼114 Mb) [8], and *C. columbae* (∼208 Mb) [7]. However, the genome sizes of these lice species are generally small compared to other insects, presumably due to the lower content of repetitive elements or the loss of redundant genes in a simple parasitic environment. We also looked at the chromosome evolution in lice. The Amblycera species, *M. gallinae*, has 5 chromosomes, while the Ischnocera species, *C. columbae*, has 12 chromosomes [7]. The latest diverging Anoplura species, *P. humanus*, has 6 chromosomes [6]. This suggests that the chromosome number in lice is highly variable, indicating potential chromosomal fission or fusion events during lice evolution. At the contig level, the contig N50 of *M. gallinae* is higher compared with other lice species, and the genome completeness estimated using BUSCO is also better (Table 2). These results indicate a well-assembled genome with a high degree of completeness and accuracy.

### Hematophagy, digestion, detoxification, and immunity-related gene families are conserved across lice genomes

In general, host-switching between birds and mammals occurred very early in the diversification of lice, and the ancestor of Afrotheria (elephants, elephant shrews, and hyraxes) acquired these parasites via host-switching from an ancient avian host [9, 11]. After host-switching, many lice change specific morphological characteristics and behaviors, corresponding to adaptations to different hosts. Bird lice feed on keratin tissues such as feathers typically. Most keratins possess a complex protein secondary structure, making them hard to be digested. In contrast, mammal lice feed on blood, which possesses relatively simple defense chemistry [86–88]. However, although the host types and feeding habits changed during the host-switching process, our results revealed a general similarity in the number of digestive enzymes, detoxifying enzymes, and immunity-related genes in both bird and mammal lice.

For mammal lice, sucking from blood vessels could provide nutritional benefits but also lead to potential harms caused by pro-oxidant molecules such as heme and iron. The mammal lice may have evolved adaptations to protect themselves from iron and heme-related damage, as observed in blood-feeding arthropods [89, 90]. Hematophagy-related genes are among the slowest-evolving gene categories in sequence divergence, suggesting that they are highly conserved (Fig. 7A). However, 7 hematophagy-related genes were positively selected in mammal lice (Supplementary Table S20). These genes were crucial to heme synthesis and iron transport [59, 60, 91]. For example, coproporphyrinogen III oxidase is an enzyme crucial to the biosynthesis of heme necessary for cellular respiration and protein function [92], and mitoferrin protein transports iron into mitochondria for cellular processes like heme production and ATP synthesis [93]. We speculated that these hematophagy-related genes may be associated with their adaptation to a blood-sucking lifestyle after host-switching from birds to mammals.

### Temperature- and chemosensory-related gene families are crucial for host-switching of lice

Environmental stressors, such as high/low temperatures, can easily affect the survival, growth, and development of insects [94, 95]. Insects use various mechanisms to tolerate high temperatures, but these come at a cost to their energy and fitness levels. This can lead to reduced survival, fecundity, body size, and mating success [96, 97]. Parasitic lice can only survive for a limited time when away from host, and thus they are highly sensitive to changes in body surface temperature of host [1, 2]. According to our analyses, mammal lice have fewer temperature-related genes compared to bird lice, especially for Hsp60 and Hsp70 genes. Since the transition of host-switching is from birds to mammals, the loss of multiple unnecessary temperature-related genes in mammal lice might reverse energy and resources for other essential biological processes to adapt to the environment. Interestingly, however, mammal lice possess a greater number of sHsp genes than bird lice. The sHsps are the first line of cell defense, preventing irreversible denaturation of substrate proteins, especially when cells are stressed, and have critical roles in normal development in insects [98–100]. Our results indicated a potential difference in the genetic basis of temperature-related genes in bird and mammal lice.

The number of chemosensory-related genes in mammal lice has also remarkably reduced compared to bird lice. GR genes are among the fastest-evolving gene categories for both copy number variation and sequence divergence in 2 lice (Figs. 5A and 7B). Mammal lice retained only the GR28 genes related to sensing host temperature from their avian ancestors during host-switching. A previous study has reported that sugar receptor (GR5a and GR64e) and CO_2_ receptor (GR21a and GR63a) were absent in mammal lice [6]. The lack of sugar receptors is a common feature among various blood feeders, including kissing bug *R. prolixus*, the bedbug *Cimex lectularius*, and the tsetse flies, several *Glossina* species [101–103]. Interestingly, the IR25a gene, the most highly conserved olfactory receptor for CO_2_ attraction among insects, was positively selected in mammal lice. It is possible that mammal lice that lack the CO_2_ receptor still respond to CO_2_ with the same IR25a-dependent pathway [104]. Overall, after host-switching from birds to mammals, lice lose these genes with sugar, fructose, and carbon dioxide receptors.

## Conclusions

In this study, we present a high-quality chromosomal-level genome assembly of *M. gallinae* with high coverage and contiguity. The *M. gallinae* genome provides a possibility to study the details of gene selection or loss in the process of evolution and adaptation to the host-switching of lice, including genes involved in hematophagy, digestion, chemosensory systems, temperature, immunity, and detoxification. Our comparative analyses have revealed genetic variations of parasitic lice, which likely correlated with host-switching from birds to mammals. We observed contractions in chemosensory and temperature-related gene families and discovered 7 hematophagy-related genes were positively selected in mammal lice. This study offers valuable genomic resources and insights into the genetic basis of *M. gallinae* and facilitates further studies on how parasitic lice adapt to host-switching. To confirm the findings of this study and determine the biological significance of relevant genes, broader genomic studies that include high-quality genome assemblies of more species and functional evidence based on experimental verification will be necessary.

## Additional Files

**Supplementary Fig. S1**. Comparison of digestive enzyme genes in *Menopon gallinae, Pediculus humanus*, and other species. Different types of digestive enzyme genes are represented by different colors. The number and proportion of serine protease genes are labeled within the green bar and the total number of digestive enzyme genes is shown next to the bar graph in each species.

**Supplementary Fig. S2**. Phylogenetic relationships of *Menopon gallinae* (MG) (A) gustatory receptor (GR) and (B) odorant receptor (OR) gene families in comparison with *Drosophila melanogaster* (DM) and *Pediculus humanus* (Phum).

**Supplementary Fig. S3**. (A) Numbers of detoxification genes among *Drosophila melanogaster, Menopon gallinae*, and *Pediculus humanus*. (B) The location of cytochrome P450 genes on chromosomes of *Menopon gallinae*. The 10 expanded CYP3 genes are clustered and highlighted in the red box.

**Supplementary Table S1**. The statistics of gene count, mRNA count, CDS count, exon count, and intron count in the genomes of *Menopon gallinae*.

**Supplementary Table S2**. The statistics of annotation in the genomes of *Menopon gallinae*.

**Supplementary Table S3**. The statistics of repeat contents in the genomes of *Menopon gallinae*.

**Supplementary Table S4**. The number of 4 categories orthologous in *Menopon gallinae, Pediculus humanus*, and other 8 species.

**Supplementary Table S5**. GO enrichment analysis of expanded genes of *Menopon gallinae*.

**Supplementary Table S6**. KEGG enrichment analysis of expanded genes of *Menopon gallinae*.

**Supplementary Table S7**. GO enrichment analysis of contracted genes of *Menopon gallinae*.

**Supplementary Table S8**. KEGG enrichment analysis of contracted genes of *Menopon gallinae*.

**Supplementary Table S9**. GO enrichment analysis of expanded genes of *Pediculus humanus*.

**Supplementary Table S10**. GO enrichment analysis of contracted genes of *Pediculus humanus*.

**Supplementary Table S11**. GO enrichment analysis of contracted genes of parasitic lice.

**Supplementary Table S12**. KEGG enrichment analysis of contracted genes of parasitic lice.

**Supplementary Table S13**. GO enrichment analysis of expanded genes of *Liposcelis brunnea*.

**Supplementary Table S14**. KEGG enrichment analysis of expanded genes of *Liposcelis brunnea*.

**Supplementary Table S15**. GO enrichment analysis of specific genes of *Menopon gallinae*.

**Supplementary Table S16**. KEGG enrichment analysis of specific genes of *Menopon gallinae*.

**Supplementary Table S17**. GO enrichment analysis of specific genes of *Pediculus humanus*.

**Supplementary Table S18**. GO enrichment analysis of specific genes of *Liposcelis brunnea*.

**Supplementary Table S19**. KEGG enrichment analysis of specific genes of *Liposcelis brunnea*.

**Supplementary Table S20**. Positively selected analysis of *Pediculus humanus*.

**Supplementary Table S21**. The number of chemosensory proteins, heat shock proteins, digestive enzyme, and immunity-related genes in the genomes of *Menopon gallinae, Pediculus humanus*, and other 8 species.

**Supplementary Table S22**. The number of detoxification gene families in the genomes of *Menopon gallinae, Pediculus humanus*, and *Drosophila melanogaster*.

## Data Availability

Bioproject and biosample for the genomic data of *M. gallinae* were submitted to NCBI under accession numbers PRJNA939264/SAMN33461892. PacBio HiFi, Illumina, Hi-C, Iso-seq, and RNA-seq data have been submitted to NCBI SRA under accession numbers SRR23634153, SRR23634151, SRR23634152, SRR23634150, and SRR23634149. The final chromosome-level genome assembly of *M. gallinae* has been submitted to the NCBI Genome under accession number JARGDH000000000. All additional supporting data are available in the *GigaScience* repository, GigaDB [105].

## Abbreviations

BLAST: Basic Local Alignment Search Tool; BUSCO: Benchmarking Universal Single-Copy Orthologs; CDS: coding sequence; GO: Gene Ontology; GR: gustatory receptors; Hsp: heat shock protein; IR: ionotropic receptor; KEGG: Kyoto Encyclopedia of Genes and Genomes; LTR: long terminal repeat; NCBI: National Center for Biotechnology Information; OG: orthogroup; OR: odorant receptor; PCG: protein-coding gene; RNA-seq: RNA sequencing; TE: transposable element; TR: tandem repeat; COI: cytochrome c oxidase subunit Ⅰ; Mya: million years ago; FDR: false discovery rate; sHsp: small heat shock protein; PGRP: peptidoglycan recognition proteins; GNBP: gram-negative bacteria binding protein.

## Competing Interests

The authors declare that they have no competing interests.

## Funding

This study was supported by the National Natural Science Foundation of China (Nos. 32170474, 31922012) and the Young Elite Scientist Sponsorship Program by CAST (No. YESS20200106).

## Authors’ Contributions

F.S. and H.L. conceived and designed the study; Y.X., Y.X.L., Q.Q.L., and Z.X.H. conducted the collection and photography of the insect; Y.X. and L.M. analyzed the data; Y.X. wrote the draft manuscript; Y.X., S.L.L., L.T., Y.G.D., W.Z.C., H.L., and F.S. discussed the results amd improved and revised the manuscript. All authors reviewed the manuscript.

## Supplementary Material

giae004_Xu_Ye-Supplemental_Information-FigureS1-S3

giae004_Xu_Ye-Supplemental_Information-TableS1-S22

giae004_GIGA-D-23-00237_Original_Submission

giae004_GIGA-D-23-00237_Revision_1

giae004_GIGA-D-23-00237_Revision_2

giae004_Response_to_Reviewer_Comments_Original_Submission

giae004_Response_to_Reviewer_Comments_Revision_1 Xinhai Ye -- 9/4/2023

giae004_Reviewer_2_Report_Original_Submission Drew Sweet -- 9/21/2023

giae004_Reviewer_2_Report_Revision_1 Drew Sweet -- 11/28/2023
