# Supplementary material for: Chromosome-level genome of the poultry shaft louse Menopon gallinae provides insight into the host-switching and adaptive evolution of parasitic lice
Source: Gigascience. 2024 Feb 19;13:giae004. doi: 10.1093/gigascience/giae004 (PMC10904027; doi:10.1093/gigascience/giae004)
Supplement: giae004_Xu_Ye-Supplemental_Information-FigureS1-S3 [file giae004_xu_ye-supplemental_information-figures1-s3.docx]

**Supplemental Information**

**Chromosome-level genome of the poultry shaft louse *Menopon gallinae* provides insight into the host-switching and adaptive evolution of parasitic lice**

Ye Xu, Ling Ma, Shanlin Liu, Yanxin Liang, Qiaoqiao Liu, Zhixin He, Li Tian, Yuange Duan, Wanzhi Cai, Hu Li^*^, Fan Song^*^

Department of Entomology and MOA Key Lab of Pest Monitoring and Green Management, College of Plant Protection, China Agricultural University, Beijing 100193, China

* **Correspondence:**

Fan Song, Email: fansong@cau.edu.cn; Hu Li, Email: [tigerleecau@hotmail.com](mailto:tigerleecau@hotmail.com)

**Supplemental Tables**

| **Table S1** The statistics of gene count, mRNA count, CDS count, exon count and intron count in the genomes of *Menopon gallinae*. |
| --- |
| **Table S2** The statistics of annotation in the genomes of *Menopon gallinae*. |
| **Table S3** The statistics of repeat contents in the genomes of *Menopon gallinae*. |
| **Table S4** The number of four categories orthologous in *Menopon gallinae*, *Pediculus humanus* and other 8 species. |
| **Table S5** GO enrichment analysis of expanded genes of *Menopon gallinae*. |
| **Table S6** KEGG enrichment analysis of expanded genes of *Menopon gallinae*. |
| **Table S7** GO enrichment analysis of contracted genes of *Menopon gallinae*. |
| **Table S8** KEGG enrichment analysis of contracted genes of *Menopon gallinae*. |
| **Table S9** GO enrichment analysis of expanded genes of *Pediculus humanus*. |
| **Table S10** GO enrichment analysis of contracted genes of *Pediculus humanus*. |
| **Table S11** GO enrichment analysis of contracted genes of parasitic lice. |
| **Table S12** KEGG enrichment analysis of contracted genes of parasitic lice. |
| **Table S13** GO enrichment analysis of expanded genes of *Liposcelis brunnea*. |
| **Table S14** KEGG enrichment analysis of expanded genes of  *Liposcelis brunnea.* |
| **Table S15** GO enrichment analysis of specific genes of *Menopon gallinae*. |
| **Table S16** KEGG enrichment analysis of specific genes of *Menopon gallinae*. |
| **Table S17** GO enrichment analysis of specific genes of *Pediculus humanus*. |
| **Table S18** GO enrichment analysis of specific genes of *Liposcelis brunnea*. |
| **Table S19** KEGG enrichment analysis of specific genes of *Liposcelis brunnea*. |
| **Table S20** Positively selected analysis of *Pediculus humanus*. |
| **Table S21** The number of chemosensory proteins, heat shock proteins, digestive enzyme and immunity-related genes in the genomes of *Menopon gallinae, Pediculus humanus* and other 8 species. |
| **Table S22** The number of Detoxification gene families in the genomes of *Menopon gallinae*, *Pediculus humanus* and *Drosophila melanogaster*. |

**Supplemental Figures**

**
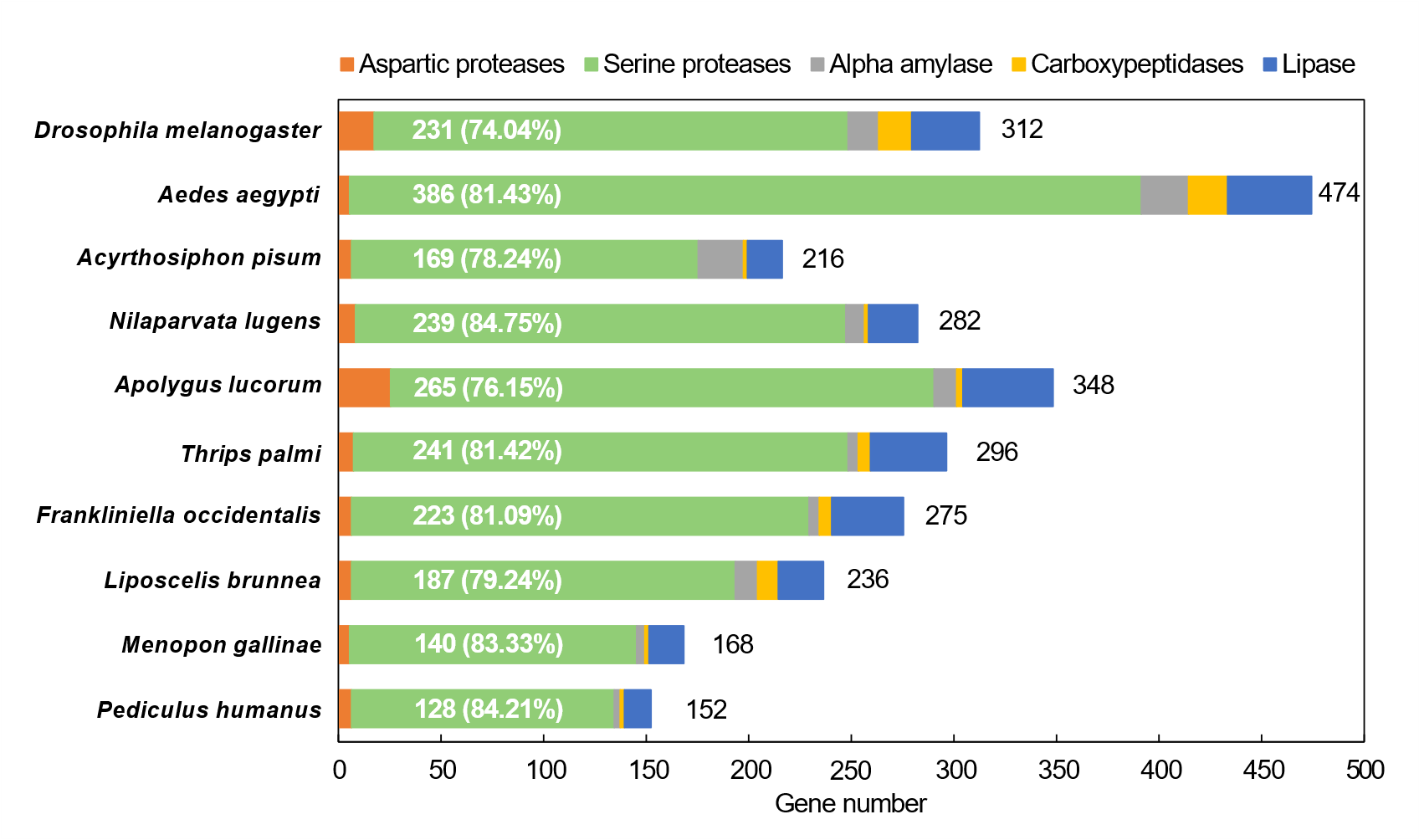
**

**Figure S1 Comparison of digestive enzyme genes in *Menopon gallinae*, *Pediculus humanus*, and other species. Different types of digestive enzyme genes are represented by different colours. The number and proportion of serine protease genes are labelled within the green bar and the total number of digestive enzyme genes is shown next to the bar graph in each species.**

**
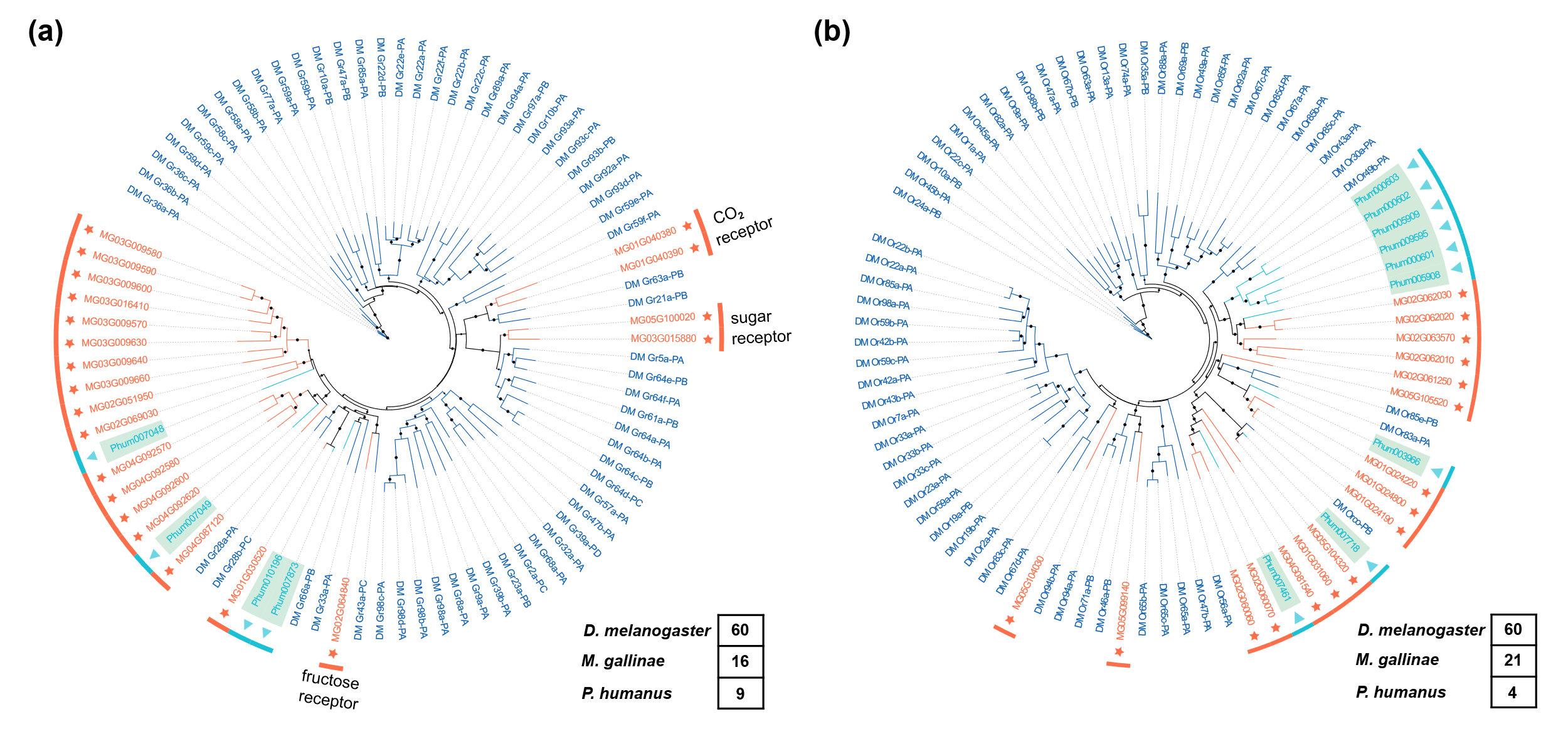
**

**Figure S2 Phylogenetic relationships of *Menopon gallinae* (MG) (a) gustatory receptor (GR) and (b) odorant receptor (OR) gene families in comparison with *Drosophila melanogaster* (DM) and *Pediculus humanus* (Phum).**


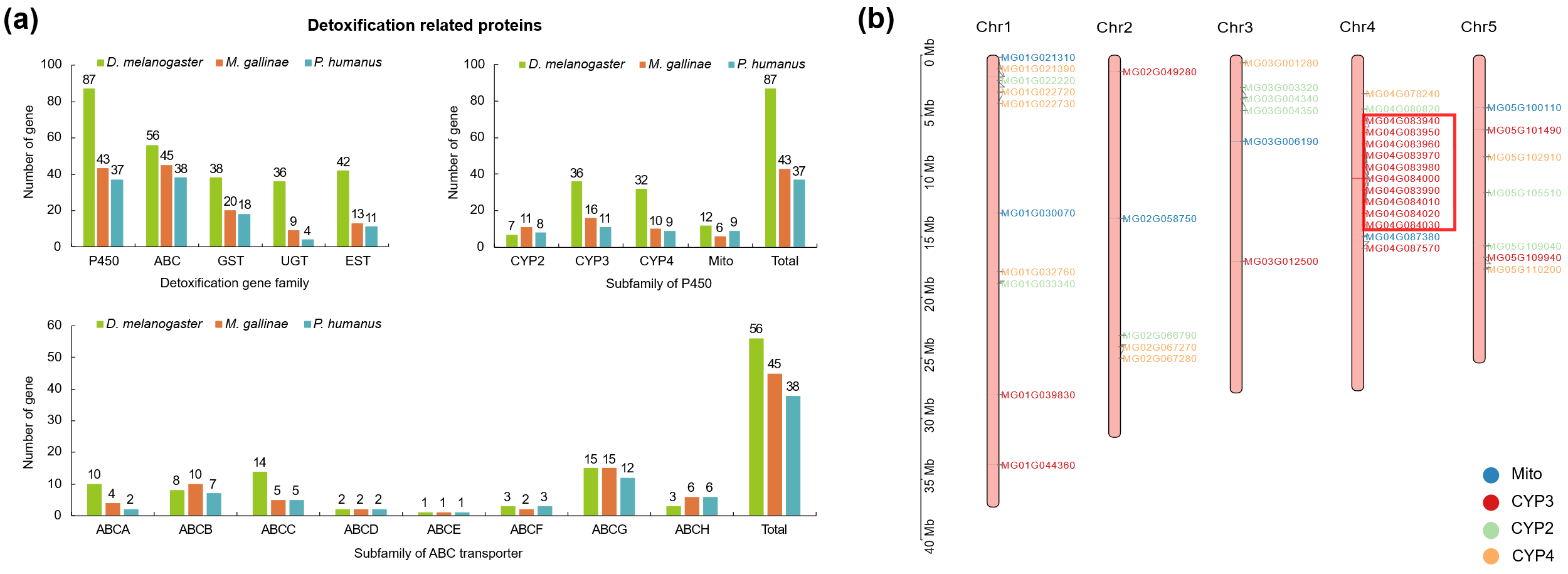


**Figure S3** (a) Numbers of detoxification genes among *Drosophila melanogaster*, *Menopon gallinae*, and *Pediculus humanus*. (b) The location of cytochrome P450 genes on chromosomes of *Menopon gallinae*. The ten expanded CYP3 genes are clustered and highlighted in the red box.
