# Supplementary material for: Chromosome-level genome of the poultry shaft louse Menopon gallinae provides insight into the host-switching and adaptive evolution of parasitic lice
Source: Gigascience. 2024 Feb 19;13:giae004. doi: 10.1093/gigascience/giae004 (PMC10904027; doi:10.1093/gigascience/giae004)
Supplement: giae004_GIGA-D-23-00237_Revision_2 [file giae004_giga-d-23-00237_revision_2.pdf]

GigaScience

Chromosome-level genome of the poultry shaft louse Menopon gallinae provides insight into the host-switching and adaptive evolution of parasitic lice

--Manuscript Draft--

Manuscript Number:	GIGA-D-23-00237R2
Full Title:	Chromosome-level genome of the poultry shaft louse Menopon gallinae provides insight into the host-switching and adaptive evolution of parasitic lice
Article Type:	Research
Funding Information:	National Natural Science Foundation of China (32170474) — Prof. Fan Song<br>National Natural Science Foundation of China (31922012) — Prof. Wanzhi Cai<br>Young Elite Scientists Sponsorship Program by CAST (YESS20200106) — Prof. Fan Song
Abstract:	Background: Lice (Psocodea: Phthiraptera) are one important group of parasites that infects birds and mammals. It is believed that the ancestor of parasitic lice originated on the ancient avian host and ancient mammals acquired these parasites via host-switching from birds. Here we present the first chromosome-level genome of Menopon gallinae in Amblycera (earliest diverging lineage of parasitic lice). We explore the transition of louse host-switching from birds to mammals at the genomic level by identifying numerous idiosyncratic genomic variations.<br>Results: The assembled genome is 155 Mb in length, with a contig N50 of 27.42 Mb. Hi-C scaffolding assigned 97% of the bases to five chromosomes. The genome of M. gallinae retains a basal insect repertoire of 11,950 protein-coding genes. By comparing the genomes of lice to those of multiple representative insects in other orders, we discovered that gene families of digestion, detoxification and immunity-related are generally conserved between bird lice and mammal lice, while mammal lice have undergone a significant reduction in genes related to chemosensory and temperature. This suggests that mammal lice have lost some of these genes through the adaption to environment and temperatures after host-switching. Furthermore, seven genes related to hematophagy were positively selected in mammal lice, suggesting their involvement in the hematophagous behavior.<br>Conclusions: Our high-quality genome of M. gallinae provides a valuable resource for comparative genomic research in Phthiraptera and facilitates further studies on adaptive evolution of host-switching within parasitic lice.
Corresponding Author:	Fan Song<br>China Agricultural University<br>Beijing, CHINA
Corresponding Author Secondary Information:	
Corresponding Author's Institution:	China Agricultural University
Corresponding Author's Secondary Institution:	
First Author:	Ye Xu
First Author Secondary Information:	
Order of Authors:	Ye Xu
	Ling Ma
	Shanlin Liu
	Yanxin Liang
	Qiaoqiao Liu

Powered by Editorial Manager® and ProduXion Manager® from Aries Systems Corporation

	Zhixin He
	Li Tian
	Yuange Duan
	Wanzhi Cai
	Hu Li
	Fan Song
**Order of Authors Secondary Information:**	
**Response to Reviewers:**	Dear Editor Hongfang Zhang:<br>Thank you for handling our article entitled “Chromosome-level genome of the poultry shaft louse Menopon gallinae provides insight into the host-switching and adaptive evolution of parasitic lice”. (ID: GIGA-D-23- 00237R1).<br>We are grateful to the positive comments of you and the reviewer. We carefully revised the issue mentioned in the reviewer's comments. Please see our point-to-point response below.<br><br>Point-to-point response:<br>Reviewer #2: Comments:<br><br>Thank you for your thorough response to my comments! I think you improved the manuscript considerably. However, I think there are a few more comments that should be addressed:<br><br>Response: We thank the reviewer for the precise comment. We have detailed our amendments below.<br><br>- Line 54: Brueelia is in Ischnocera, not Amblycera. There has been previous genomic work on Amblycera (e.g., Johnson et al. 2018), but these studies were not full genomic assemblies. Be sure to identify Brueelia as Ischnocera and refer to studies like Johnson et al. (2018) when comparing to Amblycera.<br><br>Response: Thanks for your reminder. Corrected accordingly.<br><br>- Please provide more information about the cox1 sequencing. Extraction? PCR? And how did you use the cox1 data to confirm species ID? BLAST comparisons?<br><br>Response: Thanks for the suggestion. Total genomic DNA were extracted from individual louse using the TIANamp Genomic DNA Kit (Tiangen, Beijing, China) following the manufacturer’s instructions. The COI fragments were amplified by PCR and were sequenced by Sanger sequencing. We also added description in Methods. After BLAST comparison, we confirm these COI sequences are from the same species. After receiving this revision, we also assembled mitogenome from the Illumina sequencing library, and this assembled COI sequence is also quite similar to COI fragments by PCR.<br><br>- Please clarify what you mean by "genome survey" with the Illumina data. Are you referring to the GenomeScope analysis?<br><br>Response: Thanks for this question and sorry for the ambiguity. The genome survey is mean to estimate the genome size, heterozygosity, and duplication of the genome using JELLYFISH version 2.1.3 and GenomeScope version 2.0. We also clarify in Methods.<br><br>- Why did you use assembly size to choose your assembly? The WTDBG2 and Flye assemblies were very close in size, but the Flye assembly had fewer contigs and a better BUSCO score.<br><br>Response: Thanks for this question. Among WTDBG2 and Flye, WTDBG2 produced a larger genome size (155 Mb) and a longer contig N50 (27.42 Mb), therefore the genome assembly from WTDBG2 was used. In the old Revised version, we have stated that: “At the contig level, we generated a final genome assembly of 155 Mb

	using WTDBG2 as this tool produced the largest contig N50 compared with other tools we tried”.<br><br>- Line 186 regarding TimeTree seems misplaced? Please edit this sentence.<br><br>Response: Corrected accordingly.<br><br>- Line 474: What do you mean by "the origin of the individuals was pure?" I think better to rephrase to reinforce your point that your pooled samples are likely genetically very similar and highly inbred (even though not intentionally inbred in a controlled environment).<br><br>Response: Thanks for the suggestion. We rephrased this sentence to reinforce our point.<br><br>- Do you cox1 data give you any insight about the genetic diversity among your samples? If cox1 is very similar among your samples, this gives you further evidence that your samples are genetically similar.<br><br>Response: According to the BLAST comparison, COI fragments are almost the same among our sequenced samples. We think this is due to our louse are collected from several chickens in the same room of the chicken farm.<br><br>- Line 479: I do not think Menopon is the earliest diverging lineage of Amblycera. Do you mean that Amblycera is sister to the rest of parasitic lice?<br><br>Response: Thanks for your reminder. We mean that Amblycera is sister to the rest of parasitic lice. We corrected this sentence.<br><br>Again, we thank you and the reviewer for the invaluable comments that have greatly improved our manuscript!<br>Sincerely yours,<br>Fan Song, Associate Professor<br>Department of Entomology<br>China Agricultural University
**Additional Information:**	
**Question**	**Response**
Are you submitting this manuscript to a special series or article collection?	No
**Experimental design and statistics**<br><br>Full details of the experimental design and statistical methods used should be given in the Methods section, as detailed in our Minimum Standards Reporting Checklist. Information essential to interpreting the data presented should be made available in the figure legends.<br><br>Have you included all the information requested in your manuscript?	Yes
**Resources**	Yes

Powered by Editorial Manager® and ProduXion Manager® from Aries Systems Corporation

A description of all resources used, including antibodies, cell lines, animals and software tools, with enough information to allow them to be uniquely identified, should be included in the Methods section. Authors are strongly encouraged to cite Research Resource Identifiers (RRIDs) for antibodies, model organisms and tools, where possible.<br><br>Have you included the information requested as detailed in our Minimum Standards Reporting Checklist?	
**Availability of data and materials**<br><br>All datasets and code on which the conclusions of the paper rely must be either included in your submission or deposited in publicly available repositories (where available and ethically appropriate), referencing such data using a unique identifier in the references and in the "Availability of Data and Materials" section of your manuscript.<br><br>Have you have met the above requirement as detailed in our Minimum Standards Reporting Checklist?	Yes

Powered by Editorial Manager® and ProduXion Manager® from Aries Systems Corporation

Manuscript Click here to access/download;Manuscript;Manuscript_Jan 14.docx

Chromosome-level genome of the poultry shaft louse *Menopon gallinae* provides insight into the host-switching and adaptive evolution of parasitic lice

Ye Xu, Ling Ma, Shanlin Liu, Yanxin Liang, Qiaoqiao Liu, Zhixin He, Li Tian, Yuange Duan, Wanzhi Cai, Hu Li*, Fan Song*

Department of Entomology and MOA Key Lab of Pest Monitoring and Green Management, College of Plant Protection, China Agricultural University, Beijing 100193, China

*** Correspondence:**

Fan Song, Email: fansong@cau.edu.cn; Hu Li, Email: tigerleecau@hotmail.com

Ye Xu [0009-0003-2337-1218];

Shanlin Liu [0000-0001-8118-8313];

Qiaoqiao Liu [0000-0003-0578-4054];

Zhixin He [0000-0002-9148-8157];

Yuange Duan [0000-0003-2311-9859];

Fan Song [0000-0002-2900-4174].

Abstract

Background: Lice (Psocodea: Phthiraptera) are one important group of parasites that infects birds and mammals. It is believed that the ancestor of parasitic lice originated on the ancient avian host and ancient mammals acquired these parasites via host-switching from birds. Here we present the first chromosome-level genome of *Menopon gallinae* in Amblycera (earliest diverging lineage of parasitic lice). We explore the transition of louse host-switching from birds to mammals at the genomic level by identifying numerous idiosyncratic genomic variations.

Results: The assembled genome is 155 Mb in length, with a contig N50 of 27.42 Mb. Hi-C scaffolding assigned 97% of the bases to five chromosomes. The genome of *M. gallinae* retains a basal insect repertoire of 11,950 protein-coding genes. By comparing the genomes of lice to those of multiple representative insects in other orders, we discovered that gene families of digestion, detoxification and immunity-related are generally conserved between bird lice and mammal lice, while mammal lice have undergone a significant reduction in genes related to chemosensory and temperature. This suggests that mammal lice have lost some of these genes through the adaption to environment and temperatures after host-switching. Furthermore, seven genes related to hematophagy were positively selected in mammal lice, suggesting their involvement in the hematophagous behavior.

Conclusions: Our high-quality genome of *M. gallinae* provides a valuable resource for comparative genomic research in Phthiraptera and facilitates further studies on adaptive evolution of host-switching within parasitic lice.

Keywords: *Menopon gallinae*, genome, comparative genomics, host-switching, parasitic lice

Introduction

Lice (Insecta: Phthiraptera) are parasites that infest birds and mammals with more than 4,500 species of chewing lice (Amblycera, Ischnocera, Trichodectera and Rhynchophthirina) and 500 species of blood-feeding sucking lice (Anoplura) [1,2]. Chewing lice feed on the feathers, sebaceous secretions, and skin of their avian and mammalian hosts [1], while sucking lice which parasitize only mammals have piercing-sucking mouthparts and feed exclusively on blood [3]. These parasites entirely rely on the body of the host and they affix their eggs to hairs or feathers of the host [1,3].

As an obligate parasite of domestic chickens (*Gallus gallus*), the poultry shaft louse *Menopon gallinae* (NCBI:txid328185) is a main vector for chicken diseases. These lice live on the skin, penetrate within the skin, or even burrow into the air sacs or under the feathers of chickens. Infestation by these lice can lead to annoyance, decreased weight gain, reduced egg production, egg abandonment in brooding hens, and chick mortality [4]. Additionally, they can cause high morbidity, which adversely affects the economic production of poultry [5].

To date, only three louse genomes have been published. Due to technical limitations, the reference genomes of human body louse *Pediculus humanus* (Anoplura) and pigeon wing louse *Columbicola columbae* (Ischnocera) were generated from hundreds or thousands of pooled individuals [6,7]. In contrast, Sweet et al. (2023) recently published a genome assembly from a single individual of feather louse *Brueelia nebulosa* (Ischnocera) [8]. Despite different strategies used for genome sequencing and assembly, all three louse genomes had high completeness, indicating the contribution of the robust and well-established bioinformatic

pipelines in facilitating the genome assembly. Compared with other insects, lice have a reduced number of protein-coding genes (PCGs), including fewer opsin genes, odorant receptors, and detoxification pathways [6,7]. Our understanding of the genomic signatures of parasitism in Phthiraptera is limited largely to these three species. The chromosome-level genome data of the chewing lice suborder Amblycera, which represents the earliest diverging group of lice, is still lacking. Notably, recent studies performed whole genome sequencing of several lice species and obtained high quality data, but these data are not assembled and annotated [9] and are not included in our analyses. In addition, a recent study presents a high-quality genome assembly of booklice *Liposcelis brunnea*, a phylogenetic sister group of the two parasitic lice. The *Liposcelis brunnea* genome is crucial in understanding the origins and evolution of parasitic lice [10]. Recent studies suggested that parasitic lice have an avian ancestral host and the ancestor of Afrotheria mammals acquired these parasites via host-switching [9,11]. After this host-switching from birds to mammals, parasitic lice have colonized other lineages of mammals through host-switching and co-diversified with their host. Parasitic lice have specific morphological and behavioral adaptations for attachment and avoiding host defenses [1,2]. In contrast to bird lice, mammal lice are morphologically adapted to live on their mammal hosts with tibial tarsal claws to attach to host hairs and highly derived mouthparts for feeding directly from host blood vessels [12].

During host-switching of lice, their genomes accumulate mutations, some of which may be directly linked to functional adaptations. Identifying such genomic feature and linking them to phenotypic differences is critical for deciphering the genomic drivers of species adaptability.

Expansion or contraction of key gene families may facilitate the emergence of novel functions, leading to successful host-switching of lice. Therefore, it is necessary to reveal significant variations in the genome during the host-switching of parasitic lice from birds to mammals.

In this study, we presented a high-quality chromosome-level genome of *M. gallinae* (representing the earliest diverged lice Amblycera) using a combination of Illumina short-read sequencing, PacBio high-fidelity (HiFi) long-read sequencing, and Hi-C technology. Combining the genome of human body louse *P. humanus*, the latest diverged lice (Anoplura), together with other various representative insect species, we performed comparative genomic analyses to evaluate the evolution of genes putatively involved in host-switching of parasitic lice from birds to mammals. These data would supply a useful genetic resource for future research of parasitic lice.

Methods

Samples collection and identification

For genome sequencing of the poultry shaft louse *Menopon gallinae*, approximately 1600 individuals were collected from natural populations infesting chickens (*Gallus gallus*) in Chongqing, China. The 1600 individuals were simultaneously collected from the same chicken farm. Species identification was determined using a combination of morphological identification under the microscope according to Price et al. (2003) [1] and molecular identification through sequencing of COI fragments (~550 bp).

DNA extraction, RNA extraction, library construction and sequencing

Total genomic DNA was extracted from individual louse using the TIANamp Genomic DNA Kit (Tiangen, Beijing, China) following the manufacturer's instructions. The COI were amplified by polymerase chain reaction (PCR) and the PCR products were sequenced by Sanger sequencing at Tsingke Biotechnology (Beijing, China).

Genomic DNA used for the SMRTbell library preparation was extracted from about 1,000 adults with the Blood & Cell Culture DNA Midi Kit (Qiagen, Hilden, Germany). After assessing the quality of the isolated DNA, a ~20-kb library was constructed using the SMRTbell Express Template Prep Kit 2.0 (Pacific Biosciences, California, USA). HiFi long clean reads produced by circular consensus sequencing (CCS) on the PacBio Sequel II platform were used for contig-level genome assembly.

For genome survey, genomic DNA was extracted from 50 adults and an Illumina sequencing library was constructed according to the manufacturer's instructions (Illumina, California, USA). The library was then sequenced on the Illumina NovaSeq 6000 platform in paired-end 150-bp mode to generate approximately 50 Gb data.

For genome annotation, total RNA was extracted from 50 adults using the Tiangen RNA extraction kit (Beijing, China). After reverse transcription of mRNA into cDNA, another Illumina RNA-seq library was constructed and sequenced with the same parameters, generating approximately 6 Gb data. In addition, a PacBio Iso-Seq library was constructed using the SMRTbell Express Template Prep Kit 2.0 (Pacific Biosciences, California, USA) from 50 adults and sequenced on the PacBio Sequel II platform (RRID:SCR_017990), generating

approximately 60 Gb data.

To construct a chromosomal-level assembly of the genome, we constructed the Hi-C library. In brief, 600 adults of *M. gallinae* were immersed in 2% formaldehyde for cross-linking of cellular protein. The purified nuclei were digested with 100 units of DpnII enzyme. Then Hi-C samples were extracted by biotin labelling, flat end ligation, DNA purification and random shearing of DNA into 300-600 bp fragments. Finally, the Hi-C libraries were quantified and sequenced using the Illumina NovaSeq platform (RRID:SCR_016387) with paired-end 150-bp reads.

Genome assembly and evaluation

The Illumina reads were used for genome survey, aiming to estimate the genome size, heterozygosity, and duplication using the K-mer method. Specifically, 17-base oligonucleotide K-mers were counted using JELLYFISH (RRID:SCR_005491) version 2.1.3 [13]. The genome features were then evaluated using GenomeScope (RRID:SCR_017014) version 2.0 [14]. We used several approaches to assemble the *M. gallinae* genome. The following tools were tried: WTDBG2 (RRID:SCR_017225) version 2.5 [15], HiCanu version 2.1.1 [16], Hifiasm (RRID:SCR_021069) version 0.13 [17] and Flye (RRID:SCR_017016) version 2.9.2 [18]. The purge_dups (RRID:SCR_021173) version 1.2.6 [19] was used to remove potential haplotypic duplications and contig overlaps. The statistic details resulted from different tools were summarized in Table 2. WTDBG2 and Flye produced remarkably larger contig N50 (27.42 Mb and 27.21 Mb) compared with the other two tools (6.34 Mb and 1.25 Mb). Then, among WTDBG2 and Flye, WTDBG2 produced a larger genome size (155 Mb) and therefore the

genome assembly from WTDBG2 was used. Clean reads sequenced from the Hi-C library were aligned to the contig-level genome with an end-to-end algorithm implemented in BWA-MEM version 0.7.17 [20]. Juicer (RRID:SCR_017226) version 1.6 [21] and 3D-DNA version 180419 [22] were used to assemble the scaffolds into a chromosome-level genome. The chromosome-level genome was reviewed using Juicebox (RRID:SCR_021172) version 1.11.08 The completeness of the genome was assessed using BUSCO version 3.0.2 with the insecta_odb10 database [23].

Repeat sequences annotation

We used RepeatMasker (RRID:SCR_012954) version 4.0.7 [24] and RepeatProteinMasker version 4.0.7 [24] to identify and annotate repeat sequences based on RepBase (RRID:SCR_021169) edition 2017012732 [25]. RepeatModeler (RRID:SCR_015027) version 2.0.4 [26] was used to construct a de novo repeat library. LTR FINDER (RRID:SCR_015247) version 1.0726 [27] and LTR retriever version 2.9.028 [28] were used to identify LTR retrotransposons. Tandem Repeats Finder (TRF) (RRID:SCR_022065) version 4.09.1 [29] was used to annotate tandem repeats.

Protein-coding gene annotation

We used three kinds of evidence to annotate PCGs, including ab initio, RNA-seq-based, and homolog-based methods. For RNA-seq-based gene prediction, we mapped short reads of *M. gallinae* from Illumina transcriptome sequencing to the genome using HISAT version 2.2.1 [30]. The mapped reads were used to assemble transcripts with StringTie (RRID:SCR_016323) version 2.4.0 [31]. The IsoSeq data was also processed using IsoSeq3

(RRID:SCR_022749)version 3.8.2 with certain parameters like filtering, clustering, and polishing. The two transcripts were chosen as mRNA evidence. For the homolog-based approach, we downloaded protein sequences of ten species (*Drosophila melanogaster* [32], *P. humanus* [6], *C. columbae* [7], *Bombyx mori* [33], *Acyrthosiphon pisum* [34], *Tribolium castaneum* [35], *Anopheles gambiae* [36], *Acromyrmex echinatior* [37], *Apis mellifera* [38]and *Nasonia vitripennis* [39]) from NCBI and InsectBase 2.0 [40]. For the ab initio method, we used Exonerate version 2.4.0 [41] to align homologous proteins and transcripts. Additionally, we utilized the bam2hints program in AUGUSTUS (RRID:SCR_008417) version 3.2.3 [42] to transfer the sorted and mapped bam file of RNA-seq data into a hints file. These trained gene sets and hint files were then combined as inputs for AUGUSTUS version 3.2.3 [42] to predict coding genes from the assembled genome. Finally, the high-confidence gene set was generated by merging ab initio, RNA-seq-based, and homology-based genes using MAKER (RRID:SCR_005309) version 2.31.10 [43].

Identification of orthologous genes and inference of phylogenetic relationships

To infer the phylogenetic relationships of *M. gallinae* and other insects, we selected additional nine species for phylogenetic analysis. We utilized all the protein sequences of ten insects and selected two dipteran insects as an outgroup. OrthoFinder (RRID:SCR_017118) version 2.5.4 [44] was used to find gene families including single copy genes and paralogous gene families. Based on the results of OrthoFinder, gene family clusters were divided into five categories, (1) single-copy genes in all species, (2) multiple-copy genes in at least one species, (3) species-specific genes (genes absent in other N-1 species), and (4) other genes.

The phylogenetic tree was inferred using single copy orthologues in each species. Sequence alignment was performed using MAFFT (RRID:SCR_011811) version 7.520 [45], and the resulting alignment was trimmed with the option "automated1" using trimAl version 1.4.rev15 [46]. We estimated the phylogenetic tree using the concatenated sequences of aligned proteins in IQ-TREE (RRID:SCR_017254) version 2.1.4 [47] with options "-m TEST -bb 1000 -alrt 1000". The best-fit model (Q.insect+I+G4) were compared and selected according to the Bayesian Information Criterion (BIC) by using ModelFinder [48]. The divergence time was estimated using MCMCTree (clock =3, RootAge = 4.0, rgene_gamma =1 15.83709, sigma2_gamma =1 4.5) from PAML version 4.9 [49] with the approximate likelihood method. Two calibration times based on previous studies [50–52] from the TIMETREE database (RRID:SCR_021162) were utilized for estimation: *A. pisum-P. humanus* (172.6–416.6 Mya) and *A. pisum-Apolygus lucorum* (112.5–391.7 Mya).

Gene family expansion, contraction and annotation

CAFÉ (RRID:SCR_005983) version 4.2.1 [53] was employed to examine gene family expansion and contraction among species with the results from OrthoFinder and the phylogenetic tree with divergence times as inputs. Phylogenetic tree topology and branch lengths were considered when inferring the significance of changes to gene-family size in each branch. Families with conditional P values lower than 0.05 were considered to have had a significantly accelerated rate of expansion or contraction. The results figure was analyzed using the R package GOplot (RRID:SCR_024419) version 1.0.2 [54].

For gene family annotation, we downloaded the protein sequences of corresponding gene

families in well-annotated insect species *P. humanus*, *D. melanogaster*, and *A. pisum* from the NCBI database. We searched these gene families against the protein sequences of 10 species with BLAST version 2.12.0 (evalue 1e−5) [55]. Notably, for the proteins annotated in Pfam database (RRID:SCR_004726) we confirmed the domains of this protein by HMMER (RRID:SCR_005305) version 3.0 [56]. The detected proteins in a species were regarded as presence and the undetected proteins were regarded as absence. For the putative lost genes in species, we used Exonerate (RRID:SCR_016088) version 2.4.0 [41] to search those protein sequences in other insects against the genome. If the sequence was not mapped to the genome, this might be a gene loss. If the target protein sequence was not mapped to the genome, or aligned to the genome but the genome sequence does not have a complete gene structure (which indicates a pseudogene), then this protein is considered to be lost in this species. Finally, each candidate gene was manually inspected and divided into subfamilies. In summary, hematophagy-related genes included iron/heme binding, transport and metabolism, oxidative stress, urea cycle enzymes, and other genes. Chemosensory gene families included gustatory receptors (GRs), odorant-binding proteins (OBPs), chemosensory proteins (CSPs), odorant receptor (ORs), ionotropic glutamate receptor (IR), and sensory neuron membrane protein (SNMP). Detoxification gene families included cytochrome P450 monooxygenases (P450s), glutathione-S-transferases (GSTs), esterases (ESTs), UDP-glycosyltransferases (UGTs), and ATP-binding cassette transporter (ABC transporter). Furthermore, we identified the heat shock protein (Hsp), five major digestive enzymes, and 36 immunity-related genes. Protein sequences of the annotated P450s, ABC transporter, Hsp, OR, and GR genes were aligned and trimmed

using MAFFT (RRID:SCR_011811) version 7.520 [45] and trimAl (RRID:SCR_017334) version 1.4.rev15 [46] with default parameters. Phylogenetic trees were constructed using IQ-TREE version 2.1.4 [47] with options "-m TEST -bb 1000 -alrt 1000" and visualized using the R package GGTREE (RRID:SCR_018560) version 3.3.1 [57].

Positive selection and *dN/dS* ratios analysis

We used MAFFT version 7.520 [45] to align the protein sequences, and subsequently converted the multiple protein sequence alignment and corresponding coding sequences (CDS) into a codon alignment using Pal2Nal version 14 [58]. To calculate *dN/dS* ratios across pair-wise alignments of each gene pair between bird lice *M. gallinae* and mammal lice *P. humanus*, we employed the Yn00 algorithm in PAML version 4.9 [49]. To identify potential positively selected genes (PSGs) in *M. gallinae* and *P. humanus*, we utilized the branch-site model of CodeML in PAML (RRID:SCR_014932) version 4.9 [49] with single-copy orthologs of ten insect species. Specifically, we set *M. gallinae*/*P. humanus* as the foreground branch, and the remaining species as background branches. We chose $p < 0.05$ as the significance threshold after FDR correction to identify a particular orthogroup as positively selected.

Results

Genome sequencing and assembly

We assembled a high-quality chromosome-level genome of *Menopon gallinae* by using a combination of PacBio long reads (28.51 Gb, 184-fold), Illumina short reads (50.79 Gb, 328.32-fold) and Hi-C reads (20.86 Gb, 134-fold). The genome size was estimated to be 145 Mb with

a heterozygosity rate of 0.363% by calculating the frequency with 17 k-mer analysis (Fig. 1a).

At the contig level, we generated a final genome assembly of 155 Mb using WTDBG2 as this tool produced the largest contig N50 compared with other tools we tried (see **Methods**). The genome consists of 100 contigs with an N50 of 27.42 Mb (Table 1). This final genome size of *M. gallinae* (155 Mb) is comparable to our preliminary estimation (145 Mb). However, it is larger than the previously published genome size of mammal lice *P. humanus* (108 Mb) [6] and feather louse *B. nebuosa* (114 Mb) [8], smaller than that of the pigeon wing louse *C. columbae* (208 Mb) [7] and the booklice *L. brunnea* (174 Mb) [10] genome (Table 1). Our *M. gallinae* genome possessed a remarkably higher contig N50 (27.42 Mb) compared to other published genomes of parasitic lice and booklice, including *P. humanus* (34 kb), *C. columbae* (511 kb), *B. nebuosa* (293 kb) and *L. brunnea* (1.78 Mb) (Table 1). The GC content in *M. gallinae* (41%) was slightly higher than that in *B. nebuosa* (38%), *C. columbae* (36%) and booklice *L. brunnea* (35%), and remarkably higher than that in *P. humanus* (28%), which possesses an extremely AT-rich genome [6] (Table 1). The GC content of the parasitic lice genome was highly variable. Five complete chromosomes were obtained in our assembly (N50 = 27.95 Mb), consisting of 97% of the whole genome. The number of chromosomes is comparable to *P. humanus* (6), smaller than booklice *L. brunnea* (9), and notably smaller than *C. columbae* (12) [6,7,10] (Table 1). Chromosome lengths ranges from 25.47 Mb to 37.36 Mb (Figs. 1b and 1c). Moreover, BUSCO evaluation showed high completeness and accuracy of the genome assembly, with 97.2% of genes being successfully identified, including 96.8% single-copy genes and 0.4% duplicated genes. These results justify a high-quality *M. gallinae* genome that could be used in the

downstream analyses.

Genome annotation

To predict *bona fide* protein-coding genes in the *M. gallinae* genome, we employed three different approaches: *de novo* prediction, homologous gene prediction, and RNA-seq-based prediction (see Methods for details). In total, we predicted 11,950 PCGs that were supported by three approaches. The number of PCGs in *M. gallinae* was comparable to that observed in mammal lice *P. humanus* (10,773 PCGs) [6] and feather louse *B. nebuosa* (10,938 PCGs) [8], but lower than the number reported in the genome of the pigeon wing louse *C. columbae* (13,362 PCGs) [7] and the booklice *L. brunnea* (15,543 PCGs) [10] (Table 1). This result indicated that the coding genes in two parasitic lice might have experienced an ancestral loss due to their limited habitats and simple dietary regimes compared to the wide-spread booklice *L. brunnea*.

Annotation of the 11,950 PCGs in *M. gallinae* revealed an average of 8.26 exons and 7.26 introns per gene. The average length of the mRNA transcripts was 2,763.21 bp, while the average length of the coding sequence (CDS) was 1,815.98 bp (Table S1). Functional annotation revealed that 10,664 (89.24%), 9,178 (76.80%), and 8,999 (75.31%) genes matched with proteins recorded in databases NR, SwissProt, and Pfam, respectively. Furthermore, 4,166 (34.86%) and 6,562 (54.91%) genes were successfully annotated by GO terms and KEGG pathways, respectively (Table S2).

Repeat sequences (transposable elements, TEs) only made up 4.1% of the *M. gallinae* genome. This fraction of repeats is considerably lower than observed in other published genomes of parasitic lice and booklice, such as *P. humanus* (7.3%), *C. columbae* (9.7%),

particularly *B. nebuosa* (15.1%) and *L. brunnea* (15.9%) (Table 1) [6–8,10]. For specific types of TEs, 0.01% of the *M. gallinae* genome is short interspersed nuclear elements (SINEs), 0.27% is long interspersed nuclear elements (LINEs), 0.58% is long terminal repeats (LTRs), 0.56% is DNA transposon, and the other 2.30% is tandem repeats (TRs) (Table S3). TRs are much more abundant than any other types of TEs in *M. gallinae*.

Comparative genomic analysis of bird lice and mammal lice

Ortholog identification and phylogenetic inference

To get a landscape of the evolutionary gains and losses of functional genes in two parasitic lice and understand how these evolutionary dynamics is related to the phenotypic innovations of the species, we looked for orthologous genes in *M. gallinae* (representing the earliest diverged lice Amblycera, bird lice), *P. humanus* (representing the latest diverged lice Anoplura, mammal lice), and other eight representative insect species. The gene family clusters were divided into four categories, single-copy genes, multiple-copy genes, species-specific genes (unique genes), and other genes. A phylogenetic tree generated using single-copy orthologous genes showed that all species of Paraneoptera (Psocodea, Hemiptera, and Thysanoptera) formed a clade. A total of 137,947 genes from all ten species were clustered into 16,518 unique gene families (orthogroups, OGs) using OrthoFinder (see Methods). Particularly, there were 115 orthogroups specific to bird lice, while only 23 orthogroups were specific to mammal lice (Fig. 2; Table S4).

Expansion, contraction and positively selected of gene families

We used CAFÉ version 4.2.1 [53] to study the expansions and contractions of gene

families during the evolution of parasitic lice. Compared to the common ancestor of the parasitic lice, we found 559 expanded and 1,166 contracted gene families in bird lice and 447 expanded and 1,046 contracted gene families in mammal lice. Similarly, 1,029 expanded and 901 contracted gene families were founded in booklice compared to the ancestral node of booklice and parasitic lice (Fig. 2). Enrichment analysis of GO and KEGG revealed that expanded gene families in bird lice are enriched in obsolete drug binding, drug metabolism-other enzymes and Hippo signaling pathway (Fig. 3a; Tables S5 and S6), while contracted gene families are enriched in chemosensory behavior, cytochrome P450, digestive system and immune system (Fig. 3b; Tables S7 and S8). Correspondingly, for mammal lice, while the expanded gene families are enriched in negative regulation of neurogenesis, positive regulation of nitrogen compound metabolic process (Fig. 3c; Table S9), those contracted gene families are enriched in G protein-coupled receptor activity, obsolete drug binding and signaling receptor activity (Fig. 3d; Table S10). The fact that the contracted gene families of chemosensory-related pathways in two parasitic lice implies that these genomic features may be relevant to their parasite behavior after split from their common ancestor. Accordingly, we estimated the expanded and contracted gene families in the ancestor of two parasitic lice and found that the contracted genes are enriched in response to xenobiotic stimulus, response to insecticide, defense response, cytochrome P450 and digestive system (Table S11, Table S12). Next, our analysis on booklice revealed that the expanded gene families are enriched in response to xenobiotic stimulus, lectins and drug metabolism-cytochrome P450 (Table S13, Table S14).

To understand whether the species-specific genes among the three lice contribute to their

distinct behavior, we looked for the gene families present in one lice species but absent in all other nine insect species we used. We uncovered 115, 23, and 198 species-specific gene families in bird lice, mammal lice, and booklice, respectively (Table S4). Gene enrichment analysis showed that bird lice-specific gene families are enriched in pathways of response to temperature stimulus, response to heat, and response to xenobiotic stimulus (Fig. 3e; Tables S15 and S16). Mammal lice-specific gene families are enriched in innate immune response and epidermal growth factor receptor signaling pathway (Fig. 3f; Table S17). Booklice-specific gene families are enriched in cellular response to oxygen-containing compound, digestive system, and cytochrome P450 (Table S18, Table S19). This result agrees with a previous finding that P450 genes were expanded in booklice [10].

Positive selection is an important source of evolutionary innovation and one of the major forces driving species divergence. We identify the positively selected genes in single-copy genes from these species for branch-site model analysis by Maximum Likelihood (PAML) (see Methods). We found that 135 and 201 genes were positively selected in bird and mammal lice respectively (likelihood ratio test, $P<0.05$). Seven hematophagy-related genes in mammal lice were positively selected, such as heme-related genes (coproporphyrinogen III oxidase), iron-related genes (nuclear hormone receptor, NADH ubiquinone 75kD subunit, peroxinectin like, mitoferrin, transferrin2) and salivary IP5P (Table S20), which may be due to the unique hematophagous behavior of mammal lice. Based on the above results of gene expansion/contraction and positively selected genes, we will focus on hematophagy, digestion, chemosensory, temperature, immune and detoxification gene families in the following analyses.

Hematophagy-related genes

We compared hematophagy-related genes to observe the signature change of feeding habit during host-switching. We found that proteins involved in iron/heme binding, transport and metabolism, oxidative stress, urea cycle enzymes, and other hematophagy-related genes are generally conserved in two lice genomes (Fig. 4; Table S21). Among the hematophagy-related genes in mammal lice, five were absent in bird lice: heme-related genes (ferrochelatase), iron-related genes (neverland, ND-pdsw), oxidative stress-related genes (phosphoserine phosphatase), and urea cycle enzymes (arginase). Ferrochelatase is a crucial enzyme involved in heme synthesis in insects [59]. ND-pdsw plays a key role in oxidative phosphorylation and is overrepresented in blood feeding insects [60].

Major digestive enzyme analysis

As important as hematophagy-related genes, digestive enzyme are also essential for diet of lice. The digestive enzyme of parasitic arthropods are mainly involved in the digestion of host blood and other ingested proteins present in the skin [61–63]. Feather feeding in bird lice and hematophagy in mammal lice may result in different adaptations and alterations in the composition of digestive enzymes in two lice. We found bird lice (168 genes) have more digestive enzyme than mammal lice (152 genes) (Fig. S1; Table S21). Compared to other insects or even the booklice (236 genes), digestive enzymes of two parasitic lice were substantially contracted (Fig. S1).

Genes associated with chemosensory

Genes involved in chemosensory systems, especially the OR, GR and IR subfamilies, play

a critical role in feeding, mating, and predator avoidance of insects [64,65]. We collected six subfamilies of chemosensory-related genes and the two parasitic lice have remarkably reduced chemosensory-related genes. Mammal lice have fewer chemosensory-related genes (totally 63 genes) than bird lice (106 genes) (Fig. 5a; Table S21). Bird lice have 21 GR, 16 OR and 36 IR genes, while mammal lice have only four GR, nine OR and 27 IR genes (Figs. 5a, S2a and S2b). Notably, GR genes showed the most reduction in mammal lice. GR genes primarily mediate gustation, specifically detecting sweet and bitter tastants, as well as to sense carbon dioxide (CO_2) [66–69]. Previous study has reported that some GR genes (such as sugar receptor genes and CO_2 receptor genes) were absent in mammal lice [6]. Here we confirmed that the bird lice genome encodes two sugar receptor and two CO_2 receptor. In addition, we identified a fructose receptor gene in the genome of bird lice that is absent from the protein and genome sequences of mammal lice (Fig. S2a). The remaining members of GR subfamily all belong to GR28 genes: bird lice have 16 GR28 genes while mammal lice only have four GR28 genes. GR28 are temperature sensors that can help identify hosts dependent on warmth, the strategy of which has been widely used by parasites such as tsetse flies and mosquitoes [70].

Temperature-related genes

As molecular chaperones, heat shock proteins (Hsps) play important roles in helping insects cope with various ambient stresses, such as extreme temperatures, oxidation, heavy metals, and other abiotic factors [71,72]. We have collected five Hsp subfamilies and found fewer Hsp genes in mammal lice (61 genes) than in bird lice (83 genes). The main difference is that mammal lice have ten Hsp60 and nine Hsp70 genes, while bird lice have 20 Hsp60 and 19

Hsp70 genes (Figs. 5a and 6a; Table S21). Hsp40 and Hsp90 also show higher copy numbers in bird lice than in mammal lice while only sHSP is more abundant in mammal lice (seven genes) than in bird lice (three genes) (Fig. 5a; Table S21).

Immunity-related genes

In defense against pathogens, insects rely mainly on their innate immune system [73,74]. Ninety-two immunity-related genes were identified from the mammal lice genome while 90 were identified from the bird lice genome (Fig. 5b; Table S21). All components of the Toll, JAK/STAT, and JNK pathways existed in both lice, however, several gene families involved in the humoral immune system were considerably diminished or missing in two lice genomes. In case of pathogen recognition-related genes, bird lice have three PGRP while mammal lice have only one, and the GNPB protein was absent in two lice. Several components of the Imd pathway (Imd and its adaptor protein FADD) are not found in the protein and genome sequences of bird lice, as reported in mammal lice [75]. A similar result was observed in the pea aphid *A. pisum* and kissing bug *Rhodnius prolixus*, where a more extensive loss of the Imd pathway genes purportedly allowed the development of its obligate endosymbiont [76]. Furthermore, hemocytin gene was found in mammal lice genome which is absent in bird lice.

Detoxification gene family analysis

Detoxification genes are involved in the metabolic detoxification of xenobiotics, such as plant allelochemicals and synthetic insecticides [77]. The number of detoxification genes was similar in two lice as five detoxification families (P450s, GSTs, ESTs, UGTs, and ABC) of bird lice included 43, 20, 13, 9, and 45 genes, whereas mammal lice had 37, 18, 11, 4, and 38 genes

for each family (Fig. S3a; Table S22). When mapping 43 P450 genes to the chromosomes of bird lice, one gene cluster with ten CYP3 genes was found on chromosome 4 (Figs. 6b and S3b), consistent with the expansion of ten CYP3 genes (Table S7). This expansion of CYP3 has been associated with pesticide resistance and xenobiotic metabolism, as studied in several dipteran and lepidopteran insects [78,79]. For ABC transporter, only ABCH subfamilies is slightly more abundant in two lice (6 genes) than in *D. melanogaster* (3 genes) (Figs. 6c and S3a; Table S22), while ABCG5 gene was positively selected in mammal lice (Table S20).

dN/dS **ratios analysis**

We calculated the *dN/dS* ratios (between mammal and bird lice) of hematophagy, chemosensory, detoxification, temperature, digestion, and immunity-related genes with those of other gene families. Higher *dN/dS* represents faster evolution rate. We found that chemosensory genes exhibit slightly higher *dN/dS* ratios than other gene categories (p<0.001, t-test) (Fig. 7a), suggesting the rapid evolution of chemosensory genes in lice. Among chemosensory genes, GR genes have higher *dN/dS* ratios than other subfamilies (Fig. 7b), suggesting that even among the fast-evolving chemosensory genes, the GR subfamily is under relaxed selective constraints. The *dN/dS* ratios of hematophagy-related genes are significantly lower than those of chemosensory genes and other genes (p<0.001 in both comparisons, t-test) but show no significant difference with the remaining groups (Fig. 7a). Among these hematophagy-related genes, iron-related genes have the lowest *dN/dS* ratios than other genes (Fig. 7b), indicating that conservation is more evident across hematophagy-related genes.

Discussion

Successful sample collection and the chromosome-level genome assembly of *Menopon gallinae*

In this study, we present the first chromosome-level genome of Amblycera (*Menopon gallinae*) following previous genomic on Amblycera which were not full genomic assemblies [9]. Genome assembly is typically challenged by high heterozygosity and replication, particularly in small insects that require the extraction of DNA from multiple individuals to construct sequencing libraries [6,7]. Many lice are difficult to obtain due to their low abundance on a single host (usually < 10 individuals) [80]. In this study, we collected a large number of adults *M. gallinae* from a chicken farm in Chongqing and used a long-read sequencing strategy (PacBio HiFi and Hi-C) to assemble its genome. This strategy has been shown to produce high integrity and continuity in genome assembly [81–83], making it suitable for high-quality *de novo* assembly of abundant small parasitic lice genomes. Although our *Menopon gallinae* individuals are not from inbred population, they were simultaneously collected from several chickens in the same room of the chicken farm. Therefore, the lice on the hosts are likely to have a highly homogeneous genetic background. Accordingly, the heterozygosity in our samples was as low as 0.363%, the level of which was lower than that of feather louse *B. nebuosa* (1.2%) [8] (only one individual was used), melon thrips *Thrips palmi* (1.32%) [84] and mirid predator *Cyrtorhinus lividipennis* (1.7%) [85]. In addition, it's worth noting that the low *dN*/*dS* ratios in both important gene families of *M. gallinae* and *P. humanus* indicate that they have undergone strong purifying selection, meaning that the majority of deleterious mutations

have been eliminated. Although not intentionally inbred population in a controlled environment of *M. gallinae* were used to conduct genome sequencing and assembly, the pooled samples are genetically similar and highly inbred. The genome size of *M. gallinae* (155 Mb) is intermediate between the genome sizes of other lice species, including *P. humanus* (~110 Mb) [6], *B. nebuosa* (~114 Mb) [8] and *C. columbae* (~208 Mb) [7]. However, the genome sizes of these lice species are generally small compared to other insects, presumably due to the lower content of repetitive elements or the loss of redundant genes in a simple parasitic environment. We also looked at the chromosome evolution in lice. The Amblycera species, *M. gallinae*, has five chromosomes, while the Ischnocera species, *C. columbae*, has 12 chromosomes [7]. The latest diverging Anoplura species, *P. humanus*, has six chromosomes [6]. This suggests that the chromosome numbers in lice is highly variable, indicating potential chromosomal fission or fusion events during lice evolution. At the contig-level, the contig N50 of *M. gallinae* is higher in compared with other lice species, and the genome completeness estimated using BUSCO is also better (Table 1). These results indicate a well-assembled genome with a high degree of completeness and accuracy.

Hematophagy, digestion, detoxification and immunity-related gene families are conserved across lice genomes

In general, host-switching between birds and mammals occurred very early in the diversification of lice, and the ancestor of Afrotheria (elephants, elephant shrews and hyraxes) acquired these parasites via host-switching from an ancient avian host [9,11]. After host-switching, many lice change specific morphological characteristics and behaviors,

corresponding to adaptations to different hosts. Bird lice feed on keratin tissues such as feathers typically. Most keratin possess complex protein secondary structure, making them hard to be digested. In contrast, mammal lice feed on blood, which possess relatively simple defense chemistry [86–88]. However, although the host types and feeding habits changed during the host-switching process, our results revealed a general similarity in the number of digestive enzymes, detoxifying enzymes, and immunity-related genes in both bird and mammal lice.

For mammal lice, sucking from blood vessels could provide nutritional benefits, meanwhile also lead to potential harms caused by pro-oxidant molecules such as heme and iron. The mammal lice may have evolved adaptations to protect themselves from iron and heme-related damage, as observed in blood-feeding arthropods [89,90]. Hematophagy-related genes are among the slowest-evolving gene categories in sequence divergence, suggesting that they are highly conserved (Fig. 7a). However, seven hematophagy-related genes were positively selected in mammal lice (Table S20). These genes were crucial to heme synthesis and iron transport [59,60,91]. For example, coproporphyrinogen III oxidase is an enzyme crucial to the biosynthesis of heme necessary for cellular respiration and protein function [92] and mitoferrin protein transports iron into mitochondria for cellular processes like heme production and ATP synthesis [93]. We speculated that these hematophagy-related genes may be associated with their adaptation to a blood-sucking lifestyle after host-switching from birds to mammals.

Temperature- and chemosensory-related gene families are crucial for host-switching of lice

Environmental stressors, such as high/low temperatures, can easily affect the survival,

growth, and development of insects [94,95]. Insects use various mechanisms to tolerate high temperatures, but these come at a cost to their energy and fitness levels. This can lead to reduced survival, fecundity, body size, and mating success [96,97]. Parasitic lice can only survive for a limited time when away from host, thus they are highly sensitive to changes in body surface temperature of host [1,2]. According to our analyses, mammal lice have fewer temperature-related genes compared to bird lice, especially for Hsp60 and Hsp70 genes. Since the transition of host-switching is from birds to mammals, the loss of multiple unnecessary temperature-related genes in mammal lice might reverse energy and resource for other essential biological processes to adapt to the environment. Interestingly, however, mammal lice possess a greater number of sHSP genes than bird lice. The sHSPs are the first line of cell defense, preventing irreversible denaturation of substrate proteins, especially when cells are stressed, and have critical roles in normal development in insect [98–100]. Our results indicated a potential difference in the genetic basis of temperature-related genes in bird and mammal lice.

The number of chemosensory-related genes in mammal lice have also remarkably reduced compared to bird lice. GR genes are among the fastest-evolving gene categories for both copy number variation and sequence divergence in two lice (Figs. 5a and 7b). Mammal lice retained only the GR28 genes related to sensing host temperature from their avian ancestors during host-switching. Previous study has reported that sugar receptor (GR5a and GR64e) and CO_2 receptor (GR21a and GR63a) were absent in mammal lice [6]. The lack of sugar receptors is a common feature among various blood feeders, including kissing bug *R. prolixus*, the bedbug *Cimex lectularius* and the tsetse flies, several *Glossina* species [101–103]. Interestingly, IR25a gene,

the most highly conserved olfactory receptor for CO_2 attraction among insects, was positively selected in mammal lice. It is possible that mammal lice that lack CO_2 receptor still respond to CO_2 use the same IR25a-dependent pathway [104]. Overall, after host-switching from birds to mammals, lice loss these genes of sugar, fructose, and carbon dioxide receptors.

Conclusions

In this study, we present a high-quality chromosomal-level genome assembly of *Menopon gallinae* with high coverage and contiguity. The *M. gallinae* genome provides a possibility to study the details of gene selection or loss in the process of evolution and adaptation to the host-switching of lice, including genes involved in hematophagy, digestion, chemosensory, temperature, immune, and detoxification. Our comparative analyses have revealed genetic variations of parasitic lice, which likely correlated with host-switching from birds to mammals. We observed contractions in chemosensory and temperature-related gene families and discovered seven hematophagy-related genes were positively selected in mammal lice. This study offers valuable genomic resources and insights into the genetic basis of *M. gallinae*, and facilitates further studies on how parasitic lice adapt to host-switching. To confirm the findings of this study and determine the biological significance of relevant genes, broader genomic studies that include high-quality genome assemblies of more species and functional evidence based on experimental verification will be necessary.

Data availability

Bioproject/biosample for the genomic data of *M. gallinae* were submitted to NCBI under

accession numbers PRJNA939264/SAMN33461892. PacBio HiFi, Illumina, Hi-C, Iso-Seq and RNA-Seq data have been submitted to NCBI SRA under accession numbers SRR23634153, SRR23634151, SRR23634152, SRR23634150 and SRR23634149. The final chromosome-level genome assembly of *M. gallinae* have been submitted to NCBI Genome under accession number JARGDH000000000. All additional supporting data are available in the *GigaScience* repository, GigaDB [105].

Competing Interests

The authors declare that they have no competing interests.

Fundings

This study was supported by the National Natural Science Foundation of China (Nos. 32170474, 31922012) and the Young Elite Scientist Sponsorship Program by CAST (No. YESS20200106).

Author Contributions

F. Song and H. Li conceived and designed the study; Y. Xu, Y.X. Liang, Q.Q. Liu and Z.X. He conducted the collection and photography of the insect; Y. Xu and L. Ma analyzed the data; Y. Xu wrote the draft manuscript; Y. Xu, S.L. Liu, L. Tian, Y.G. Duan, W.Z. Cai, H. Li and F. Song discussed the results, improved and revised the manuscript. All authors reviewed the manuscript.

Acknowledgements

We thank Jiajun Chu at chicken farm (Chongqing, China) for help obtain samples of lice. We thank Tianyou Zhao for assisting in the analysis of the data. We thank the 2115 Talent Development Program of the China Agricultural University. We sincerely thank the editors and reviewers for their valuable suggestions and comments on this study.

References

1. Price RD, Hellenthal RA, Palma RL, Johnson KP, Clayton DH. *The Chewing Lice: World Checklist and Biological Overview*. Illinois Natural History Survey. Special Publication. Illinois; 2003.

2. Durden LA, Musser GG. The sucking lice (Insecta, Anoplura) of the world: a taxonomic checklist with records of mammalian hosts and geographical distributions. *Bull Am Museum Nat History* 1994;**218**:1–90.

3. Clayton DH, Bush SE, Johnson KP. *Coevolution of Life on Hosts: Integrating Ecology and History*. University of Chicago Press. Chicago; 2015.

4. Urquhart GM, Armour J, Duncan JL, Dunn AM, Jennings FW. *Veterinary Parasitology*. Longman Scientific and Technical. UK; 1987.

5. Pavlovic I, Blazin V, Hudina V, Ilic Z, Miljkovic B. Effect of the biting louse *Menacanthus stramineus* on reducing the egg production of poultry under intensive conditions. *Vet Glas* 1989;**43**:181–186.

6. Kirkness EF, Haas BJ, Sun W, Braig HR, Perotti MA, Clark JM, et al. Genome sequences of the human body louse and its primary endosymbiont provide insights into the permanent parasitic lifestyle. *Proc Natl Acad Sci USA* 2010;**107**(27):12168–12173.

7. Baldwin-Brown JG, Villa SM, Vickrey AI, Johnson KP, Bush SE, Clayton DH, et al. The assembled and annotated genome of the pigeon louse *Columbicola columbae*, a model ectoparasite. *G3* 2021;**11**(2):jkab009.

8. Sweet AD, Browne DR, Hernandez AG, Johnson KP, Cameron SL. Draft genome assemblies of the avian louse *Brueelia nebulosa* and its associates using long-read sequencing from an individual specimen. *G3* 2023;**13**(4)jkad030.

9. Johnson KP, Nguyen N, Sweet AD, Boyd BM, Warnow T, Allen JM. Simultaneous radiation of bird and mammal lice following the K-Pg boundary. *Biol Lett* 2018;**14**(5):20180141.

10. Feng S, Opit G, Deng W, Stejskal V, Li Z. A chromosome-level genome of the booklouse, *Liposcelis brunnea*, provides insight into louse evolution and environmental stress adaptation. *GigaScience* 2022;**11**:giac062.

11. Johnson KP, Matthee C, Doña J. Phylogenomics reveals the origin of mammal lice out of Afrotheria. *Nat Ecol Evol* 2022. doi:10.1038/s41559-022-01803-1.

12. Snodgrass RE. The feeding apperatus of biting and sucking insects affecting man and animals. *Smithson Misc Collect* 1944;**104**:1–113.

13. Marçais G, Kingsford C. A fast, lock-free approach for efficient parallel counting of occurrences of k-mers. *Bioinformatics* 2011;**27**(6):764–770.

14. Vurture GW, Sedlazeck FJ, Nattestad M, Underwood CJ, Fang H, Gurtowski J, et al. GenomeScope: fast reference-free genome profiling from short reads. *Bioinformatics* 2017;**33**(14):2202–2204.

15. Ruan J, Li H. Fast and accurate long-read assembly with wtdbg2. *Nat Methods* 2020;**17**(2):155–158.

16. Nurk S, Walenz BP, Rhie A, Vollger MR, Logsdon GA, Grothe R, et al. HiCanu: accurate assembly of segmental duplications, satellites, and allelic variants from high-fidelity long reads. *Genome Res* 2020;**30**(9):1291–1305.

17. Cheng H, Concepcion GT, Feng X, Zhang H, Li H. Haplotype-resolved de novo assembly using phased assembly graphs with hifiasm. *Nat Methods* 2021;**18**(2):170–175.

18. Kolmogorov M, Yuan J, Lin Y, Pevzner PA. Assembly of long, error-prone reads using repeat graphs. *Nat Biotechnol* 2019;**37**(5):540–546.

19. Guan D, McCarthy SA, Wood J, Howe K, Wang Y, Durbin R. Identifying and removing haplotypic duplication in primary genome assemblies. *Bioinformatics* 2020;**36**(9):2896–2898.

20. Li H, Durbin R. Fast and accurate short read alignment with Burrows-Wheeler transform. *Bioinformatics* 2009;**25**(14):1754–1760.

21. Durand NC, Shamim MS, Machol I, Rao SSP, Huntley MH, Lander ES, et al. Juicer provides a one-click system for analyzing loop-resolution Hi-C experiments. *Cell Syst* 2016;**3**(1):95–98.

22. Dudchenko O, Batra SS, Omer AD, Nyquist SK, Hoeger M, Durand NC, et al. De novo assembly of the *Aedes aegypti* genome using Hi-C yields chromosome-length scaffolds. *Science* 2017;**356**(6333):92–95.

23. Simão FA, Waterhouse RM, Ioannidis P, Kriventseva EV, Zdobnov EM. BUSCO: assessing genome assembly and annotation completeness with single-copy orthologs. *Bioinformatics* 2015;**31**(19):3210–3212.

24. Tarailo□Graovac M, Chen N. Using RepeatMasker to identify repetitive elements in

genomic sequences. *Curr Protoc Bioinformatics* 2009;**25**(1). doi:10.1002/0471250953.bi0410s25.

25. Jurka J, Kapitonov VV, Pavlicek A, Klonowski P, Kohany O, Walichiewicz J. Repbase Update, a database of eukaryotic repetitive elements. *Cytogenet Genome Res* 2005;**110**(1–4):462–467.

26. Flynn JM, Hubley R, Goubert C, Rosen J, Clark AG, Feschotte C, et al. RepeatModeler2 for automated genomic discovery of transposable element families. *Proc Natl Acad Sci USA* 2020;**117**(17):9451–9457.

27. Ou S, Jiang N. LTR_FINDER_parallel: parallelization of LTR_FINDER enabling rapid identification of long terminal repeat retrotransposons. *Mobile DNA* 2019;**10**(1):48.

28. Ou S, Jiang N. LTR_retriever: a highly accurate and sensitive program for identification of long terminal repeat retrotransposons. *Plant Physiol* 2018;**176**(2):1410–1422.

29. Benson G. Tandem repeats finder: a program to analyze DNA sequences. *Nucleic Acids Res* 1999;**27**(2):573–580.

30. Kim D, Langmead B, Salzberg SL. HISAT: a fast spliced aligner with low memory requirements. *Nat Methods* 2015;**12**(4):357–360.

31. Pertea M, Kim D, Pertea GM, Leek JT, Salzberg SL. Transcript-level expression analysis of RNA-seq experiments with HISAT, StringTie and Ballgown. *Nat Protoc* 2016;**11**(9):1650–1667.

32. Adams MD, Celniker SE, Holt RA, Evans CA, Gocayne JD, Amanatides PG, et al. The genome sequence of *Drosophila melanogaster*. *Science* 2000;**287**(5461):2185–2195.

33. Lu F, Wei Z, Luo Y, Guo H, Zhang G, Xia Q, et al. SilkDB 3.0: visualizing and exploring multiple levels of data for silkworm. *Nucleic Acids Res* 2020;**48**(D1):D749–D755.

34. Mathers TC, Wouters RHM, Mugford ST, Swarbreck D, van Oosterhout C, Hogenhout SA. Chromosome-scale genome assemblies of aphids reveal extensively rearranged autosomes and long-term conservation of the X chromosome. *Mol Biol Evol* 2021;**38**(3):856–875.

35. Kim HS, Murphy T, Xia J, Caragea D, Park Y, Beeman RW, et al. BeetleBase in 2010: revisions to provide comprehensive genomic information for *Tribolium castaneum*. *Nucleic Acids Res* 2010;**38**(suppl_1):D437–D442.

36. Sharakhova MV, Hammond MP, Lobo NF, Krzywinski J, Unger MF, Hillenmeyer ME, et al. Update of the *Anopheles gambiae* PEST genome assembly. *Genome Biol* 2007;**8**(1):R5.

37. Nygaard S, Zhang G, Schiøtt M, Li C, Wurm Y, Hu H, et al. The genome of the leaf-cutting ant *Acromyrmex echinatior* suggests key adaptations to advanced social life and fungus farming. *Genome Res* 2011;**21**(8):1339–1348.

38. Wallberg A, Bunikis I, Pettersson OV, Mosbech M-B, Childers AK, Evans JD, et al. A hybrid de novo genome assembly of the honeybee, *Apis mellifera*, with chromosome-

length scaffolds. *BMC Genomics* 2019;**20**(1):275.

39. Dalla Benetta E, Antoshechkin I, Yang T, Nguyen HQM, Ferree PM, Akbari OS. Genome elimination mediated by gene expression from a selfish chromosome. *Sci Adv* 2020;**6**(14):eaaz9808.

40. Mei Y, Jing D, Tang S, Chen X, Chen H, Duanmu H, et al. InsectBase 2.0: a comprehensive gene resource for insects. *Nucleic Acids Res* 2022;**50**(D1):D1040–D1045.

41. Slater GSC, Birney E. Automated generation of heuristics for biological sequence comparison. *BMC Bioinformatics* 2005;**6**(1):1–11.

42. Stanke M, Waack S. Gene prediction with a hidden Markov model and a new intron submodel. *Bioinformatics* 2003;**19**(Suppl 2):ii215–ii225.

43. Cantarel BL, Korf I, Robb SMC, Parra G, Ross E, Moore B, et al. MAKER: an easy-to-use annotation pipeline designed for emerging model organism genomes. *Genome Res* 2008;**18**(1):188–196.

44. Emms DM, Kelly S. OrthoFinder: solving fundamental biases in whole genome comparisons dramatically improves orthogroup inference accuracy. *Genome Biol* 2015;**16**(1):157.

45. Katoh K, Standley DM. MAFFT multiple sequence alignment software version 7: improvements in performance and usability. *Mol Biol Evol* 2013;**30**(4):772–780.

46. Capella-Gutierrez S, Silla-Martinez JM, Gabaldon T. trimAl: a tool for automated alignment trimming in large-scale phylogenetic analyses. *Bioinformatics* 2009;**25**(15):1972–1973.

47. Minh BQ, Schmidt HA, Chernomor O, Schrempf D, Woodhams MD, von Haeseler A, et al. IQ-TREE 2: new models and efficient methods for phylogenetic inference in the genomic era. *Mol Biol Evol* 2020;**37**(5):1530–1534.

48. Kalyaanamoorthy S, Minh BQ, Wong TKF, von Haeseler A, Jermiin LS. ModelFinder: fast model selection for accurate phylogenetic estimates. *Nat Methods* 2017;**14**(6):587–589.

49. Yang Z. PAML 4: Phylogenetic analysis by maximum likelihood. *Mol Biol Evol* 2007;**24**(8):1586–1591.

50. Smith VS, Ford T, Johnson KP, Johnson PCD, Yoshizawa K, Light JE. Multiple lineages of lice pass through the K–Pg boundary. *Biol Lett* 2011;**7**(5):782–785.

51. Wheat CW, Wahlberg N. Phylogenomic insights into the Cambrian Explosion, the Colonization of Land and the Evolution of Flight in Arthropoda. *Syst Biol* 2013;**62**(1):93–109.

52. Misof B, Liu S, Meusemann K, Peters RS, Donath A, Mayer C, et al. Phylogenomics resolves the timing and pattern of insect evolution. *Science* 2014. doi:10.1126/science.1257570.

53. De Bie T, Cristianini N, Demuth JP, Hahn MW. CAFE: a computational tool for the study of gene family evolution. *Bioinformatics* 2006;**22**(10):1269–1271.

54. Walter W, Sánchez-Cabo F, Ricote M. GOplot: an R package for visually combining expression data with functional analysis. *Bioinformatics* 2015;**31**(17):2912–2914.

55. McGinnis S, Madden TL. BLAST: at the core of a powerful and diverse set of sequence analysis tools. *Nucleic Acids Res* 2004;**32**(Web Server issue):W20-25.

56. Potter SC, Luciani A, Eddy SR, Park Y, Lopez R, Finn RD. HMMER web server: 2018 update. *Nucleic Acids Res* 2018;**46**(W1):W200–W204.

57. Yu G, Smith DK, Zhu H, Guan Y, Lam TT. GGTREE: an R package for visualization and annotation of phylogenetic trees with their covariates and other associated data. *Methods Ecol Evol* 2017;**8**(1):28–36.

58. Suyama M, Torrents D, Bork P. PAL2NAL: robust conversion of protein sequence alignments into the corresponding codon alignments. *Nucleic Acids Res* 2006;**34**(suppl_2):W609–W612.

59. Hamza I, Dailey HA. One ring to rule them all: Trafficking of heme and heme synthesis intermediates in the metazoans. *Biochim Biophys Acta Mol Cell Res* 2012;**1823**(9):1617–1632.

60. Ceesay M. A bioinformatics approach for evaluating evolutionary convergence of gene family size in hematophagous insects. *Theses, Dissertations and Culminating Projects* 2023. https://digitalcommons.montclair.edu/etd/1202.

61. Cruz CE, Fogaça AC, Nakayasu ES, Angeli CB, Belmonte R, Almeida IC, et al. Characterization of proteinases from the midgut of *Rhipicephalus* (*Boophilus*) *microplus* involved in the generation of antimicrobial peptides. *Parasit Vectors* 2010;**3**(1):63.

62. Mahmood W, Viberg LT, Fischer K, Walton SF, Holt DC. An aspartic protease of the scabies mite *Sarcoptes scabiei* is involved in the digestion of host skin and blood macromolecules. *PLoS Negl Trop Dis* 2013;**7**(11):e2525.

63. Santiago PB, de Araújo CN, Motta FN, Praça YR, Charneau S, Bastos IMD, et al. Proteases of haematophagous arthropod vectors are involved in blood-feeding, yolk formation and immunity - a review. *Parasit Vectors* 2017;**10**(1):79.

64. Eyun S, Soh HY, Posavi M, Munro JB, Hughes DST, Murali SC, et al. Evolutionary history of chemosensory-related gene families across the Arthropoda. *Mol Biol Evol* 2017;**34**(8):1838–1862.

65. Robertson HM. Molecular evolution of the major arthropod chemoreceptor gene families. *Annu Rev Entomol* 2019;**64**(1):227–242.

66. Fujii S, Yavuz A, Slone J, Jagge C, Song X, Amrein H. *Drosophila* sugar receptors in sweet taste perception, olfaction, and internal nutrient sensing. *Curr Biol* 2015;**25**(5):621–627.

67. Jones WD, Cayirlioglu P, Grunwald Kadow I, Vosshall LB. Two chemosensory receptors together mediate carbon dioxide detection in *Drosophila*. *Nature* 2007;**445**(7123):86–90.

68. Miyamoto T, Slone J, Song X, Amrein H. A fructose receptor functions as a nutrient sensor in the *Drosophila* brain. *Cell* 2012;**151**(5):1113–1125.

69. Shim J, Lee Y, Jeong YT, Kim Y, Lee MG, Montell C, et al. The full repertoire of *Drosophila* gustatory receptors for detecting an aversive compound. *Nat Commun* 2015;**6**(1):8867.

70. Ni L, Bronk P, Chang EC, Lowell AM, Flam JO, Panzano VC, et al. A gustatory receptor paralogue controls rapid warmth avoidance in *Drosophila*. *Nature* 2013;**500**(7464):580–584.

71. García-Reina A, Rodríguez-García MJ, Ramis G, Galián J. Real-time cell analysis and heat shock protein gene expression in the TcA *Tribolium castaneum* cell line in response to environmental stress conditions: RTCA and Hsps expression in the TcA cell line. *Insect Sci* 2017;**24**(3):358–370.

72. Lu K, Chen X, Liu W, Zhang Z, Wang Y, You K, et al. Characterization of heat shock protein 70 transcript from *Nilaparvata lugens* (Stål): Its response to temperature and insecticide stresses. *Pestic Biochem Physiol* 2017;**142**:102–110.

73. Aggarwal K, Silverman N. Positive and negative regulation of the *Drosophila* immune response. *BMB Rep* 2008;**41**(4):267–277.

74. Ferrandon D, Imler J-L, Hetru C, Hoffmann JA. The *Drosophila* systemic immune response: sensing and signalling during bacterial and fungal infections. *Nat Rev Immunol* 2007;**7**(11):862–874.

75. Kim JH, Min JS, Kang JS, Kwon DH, Yoon KS, Strycharz J, et al. Comparison of the humoral and cellular immune responses between body and head lice following bacterial challenge. *Insect Biochem Mol Biol* 2011;**41**(5):332–339.

76. Gerardo NM, Altincicek B, Anselme C, Atamian H, Barribeau SM, de Vos M, et al. Immunity and other defenses in pea aphids, *Acyrthosiphon pisum*. *Genome Biol* 2010;**11**(2):R21.

77. Nauen R, Bass C, Feyereisen R, Vontas J. The role of cytochrome P450s in insect toxicology and resistance. *Annu Rev Entomol* 2022;**67**(1):105–124.

78. Müller P, Warr E, Stevenson BJ, Pignatelli PM, Morgan JC, Steven A, et al. Field-caught permethrin-resistant *Anopheles gambiae* overexpress CYP6P3, a P450 that metabolises pyrethroids. *PLoS Genet* 2008;**4**(11):e1000286.

79. Wang H, Shi Y, Wang L, Liu S, Wu S, Yang Y, et al. CYP6AE gene cluster knockout in *Helicoverpa armigera* reveals role in detoxification of phytochemicals and insecticides. *Nat Commun* 2018;**9**(1):4820.

80. Johnson KP. Genomic approaches to uncovering the coevolutionary history of parasitic lice.

Life 2022;**12**(9):1442.

81. Li B, Du Z, Tian L, Zhang L, Huang Z, Wei S, et al. Chromosome□level genome assembly of the aphid parasitoid *Aphidius gifuensis* using Oxford Nanopore sequencing and Hi□C technology. *Mol Ecol Resour* 2021;**21**(3):941–954.

82. Xu H, Ye X, Yang Y, Yang Y, Sun YH, Mei Y, et al. Comparative genomics sheds light on the convergent evolution of miniaturized wasps. *Mol Biol Evol* 2021;**38**(12):5539–5554.

83. Ye X, Yang Y, Zhao C, Xiao S, Sun YH, He C, et al. Genomic signatures associated with maintenance of genome stability and venom turnover in two parasitoid wasps. *Nat Commun* 2022;**13**(1):6417.

84. Guo S, Cao L, Song W, Shi P, Gao Y, Gong Y, et al. Chromosome□level assembly of the melon thrips genome yields insights into evolution of a sap□sucking lifestyle and pesticide resistance. *Mol Ecol Resour* 2020;**20**(4):1110–1125.

85. Bai Y, Shi Z, Zhou W, Wang G, Shi X, He K, et al. Chromosome-level genome assembly of the mirid predator *Cyrtorhinus lividipennis* Reuter (Hemiptera: Miridae), an important natural enemy in the rice ecosystem. *Mol Ecol Resour* 2022;**22**(3):1086–1099.

86. Hughes J, Vogler AP. Gene expression in the gut of keratin-feeding clothes moths (Tineola) and keratin beetles (Trox) revealed by subtracted cDNA libraries. *Insect Biochem Mol Biol* 2006;**36**(7):584–592.

87. Kollien AH, Waniek PJ, Prols F, Habedank B, Schaub GA. Cloning and characterization of a trypsin-encoding cDNA of the human body louse *Pediculus humanus*. *Insect Mol Biol* 2004;**13**(1):9–18.

88. Waniek PJ, Hendgen-Cotta UB, Stock P, Mayer C, Kollien AH, Schaub GA. Serine proteinases of the human body louse (*Pediculus humanus*): sequence characterization and expression patterns. *Parasitol Res* 2005;**97**(6):486–500.

89. Hajdusek O, Sojka D, Kopacek P, Buresova V, Franta Z, Sauman I, et al. Knockdown of proteins involved in iron metabolism limits tick reproduction and development. *Proc Natl Acad Sci USA* 2009;**106**(4):1033–1038.

90. Hentze MW, Muckenthaler MU, Andrews NC. Balancing Acts: molecular control of mammalian iron metabolism. *Cell* 2004;**117**(3):285–297.

91. Tang X, Zhou B. Iron homeostasis in insects: Insights from *Drosophila* studies. *IUBMB Life* 2013;**65**(10):863–872.

92. Módis K, Ramanujam V-MS, Govar AA, Lopez E, Anderson KE, Wang R, et al. Cystathionine-γ-lyase (CSE) deficiency increases erythropoiesis and promotes mitochondrial electron transport via the upregulation of coproporphyrinogen III oxidase and consequent stimulation of heme biosynthesis. *Biochem Pharmacol* 2019;**169**:113604.

93. Chen W, Paradkar PN, Li L, Pierce EL, Langer NB, Takahashi-Makise N, et al. Abcb10

physically interacts with mitoferrin-1 (Slc25a37) to enhance its stability and function in the erythroid mitochondria. *Proc Natl Acad Sci USA* 2009;**106**(38):16263–16268.

94. Du Y. Insect heat shock proteins and their underlying functions. *J Integr Agric* 2018;**17**(5):1011.

95. King AM, MacRae TH. Insect heat shock proteins during stress and diapause. *Annu Rev Entomol* 2015;**60**(1):59–75.

96. Abram PK, Boivin G, Moiroux J, Brodeur J. Behavioural effects of temperature on ectothermic animals: unifying thermal physiology and behavioural plasticity: Effects of temperature on animal behaviour. *Biol Rev* 2017;**92**(4):1859–1876.

97. Huang L-H, Chen B, Kang L. Impact of mild temperature hardening on thermotolerance, fecundity, and Hsp gene expression in *Liriomyza huidobrensis*. *J Insect Physiol* 2007;**53**(12):1199–1205.

98. Arrigo A-P. Human small heat shock proteins: Protein interactomes of homo- and hetero-oligomeric complexes: An update. *FEBS Letters* 2013;**587**(13):1959–1969.

99. Basha E, O'Neill H, Vierling E. Small heat shock proteins and α-crystallins: dynamic proteins with flexible functions. *Trends Biochem Sci* 2012;**37**(3):106–117.

100. Raut S, Mallik B, Parichha A, Amrutha V, Sahi C, Kumar V. RNAi-mediated reverse genetic screen identified *Drosophila* chaperones regulating eye and neuromuscular junction morphology. *G3* 2017;**7**(7):2023–2038.

101. Mesquita RD, Vionette-Amaral RJ, Lowenberger C, Rivera-Pomar R, Monteiro FA, Minx P, et al. Genome of *Rhodnius prolixus*, an insect vector of Chagas disease, reveals unique adaptations to hematophagy and parasite infection. *Proc Natl Acad Sci USA* 2015;**112**(48):14936–14941.

102. Benoit JB, Adelman ZN, Reinhardt K, Dolan A, Poelchau M, Jennings EC, et al. Unique features of a global human ectoparasite identified through sequencing of the bed bug genome. *Nat Commun* 2016;**7**(1):10165.

103. Attardo GM, Abd-Alla AMM, Acosta-Serrano A, Allen JE, Bateta R, Benoit JB, et al. Comparative genomic analysis of six *Glossina* genomes, vectors of African trypanosomes. *Genome Biol* 2019;**20**(1):187.

104. van Breugel F, Huda A, Dickinson MH. Distinct activity-gated pathways mediate attraction and aversion to CO_2 in *Drosophila*. *Nature* 2018;**564**(7736):420–424.

105. Xu Y, Ma L, Liu S, Liang Y, Liu Q, He Z, et al. Supporting data for "Chromosome-level genome of the poultry shaft louse Menopon gallinae provides insight into the host-switching and adaptive evolution of parasitic lice". GigaScience Database. 2024. http://dx.doi.org/10.5524/102490.

Tables & Figures

Table 1 The genome features of four parasitic lice and one booklice

Feature	***Pediculus humanus***	***Menopon gallinae***	***Columbicola columbae***	***Brueelia nebuosa***	***Liposcelis brunnea***
Assembly level	Scaffold	Chromosome	Chromosome	Scaffold	Chromosome
Heterozygosity	-	0.363%	-	1.2%	0.268%
Survey	103-109 Mb	145 Mb	230 Mb	100 Mb	172 Mb
Genome size	108 Mb	155 Mb	208 Mb	114 Mb	174 Mb
Contig N50	34 kb	27.42 Mb	511 kb	293 kb	1.78 Mb
Scaffold N50	497 kb	27.95 Mb	17.67 Mb	637 kb	19.7 Mb
Chromosomes	6	5	12	-	9
BUSCO	95.9%	97.2%	96.4%	96.1%	97.2%
GC content%	28%	41%	36%	38%	35%
Protein-coding genes	10,773	11,950	13,362	10,938	15,543
Repetitive elements	7.3%	4.1%	9.7%	15.1%	15.9%

Table 2 Statistics for the assembly of *Menopon gallinae* using PacBio data.

Feature	WTDBG2	Hifiasm	HiCanu	Flye
Genome size	155 Mb	217 Mb	254 Mb	151 Mb
Number of contigs	100	805	424	24
Contig N50	27.42 Mb	6.34 Mb	1.25 Mb	27.21 Mb
BUSCO	97.2%	97.5%	98.1%	98.0%
GC content%	41%	41%	41%	41%

Figure Legends

Figure 1 Genome description of *Menopon gallinae*. (a) GenomeScope estimation of genome size and heterogeneity using a k-mer of 17. (b) Hi-C interaction map produced by 3D-DNA. (c) Circular representation of the chromosomes. Tracks a-d represents the distribution of chromosome karyotypes, gene density, GC density, and repeat sequences density, respectively. Densities were calculated in 100 kb windows.

Figure 2 Phylogenetic tree with the dynamic evolution of gene families among *Menopon gallinae*, *Pediculus humanus* and other species. In the left panel, blue and red numbers on the branch shows the number of expanded and contracted gene families for each clade. Pie charts beside or on each branch of the tree show the proportion of expanded (blue) and contracted (red) gene families. The black numbers are divergence times. In the right panel, the numbers of gene families (orthogroups) were shown as barplots. Orthogroups of different categories were in

different colors.

Figure 3 Enrichment analysis of gene families of different categories. KEGG pathway of expanded (a) and contracted (b) gene families of *Menopon gallinae*. GO enrichment of expanded (c) and contracted (d) gene families of *Pediculus humanus*. GO enrichment of specific gene families of *Menopon gallinae* (e) and *Pediculus humanus* (f).

Figure 4 Distribution of hematophagy-related genes in the genomes of *Menopon gallinae*, *Pediculus humanus*, and other species. The heatmap shows the numbers of hematophagy-related genes. The numbers were transformed with $\log_{10}(n+1)$.

Figure 5 Distribution of (a) chemosensory proteins, heat shock proteins, and (b) immunity-related proteins in *Menopon gallinae*, *Pediculus humanus*, and other species.

Figure 6 Phylogenetic relationships of *Menopon gallinae* (MG) (a) heat shock protein (HSP), (b) cytochrome P450 (P450) and (c) ATP binding cassette (ABC) transporter gene families in comparison with *Drosophila melanogaster* (DM) and *Pediculus humanus* (Phum).

Figure 7 Comparing the *dN*/*dS* ratios between *Menopon gallinae* and *Pediculus humanus*. (a, b) Gene families and subfamilies related to hematophagy, chemosensory, temperature, detoxification, digestive, immunity, and other gene families.

Cover_Letter Click here to access/download;Manuscript;Cover_Letter.docx

Department of Entomology
China Agricultural University
No.2 Yuanmingyuan West Road
Beijing, 100193
fansong@cau.edu.cn

December 4, 2023

Dear Editor Hongfang Zhang:

Thank you for handling our article entitled “**Chromosome-level genome of the poultry shaft louse *Menopon gallinae* provides insight into the host-switching and adaptive evolution of parasitic lice**”. (ID: GIGA-D-23- 00237R1).

We are grateful to the positive comments of you and the reviewer. We carefully revised the issue mentioned in the reviewer's comments. Please see our point-to-point response below.

Point-to-point response:

Reviewer #2: Comments:

Thank you for your thorough response to my comments! I think you improved the manuscript considerably. However, I think there are a few more comments that should be addressed:

Response: We thank the reviewer for the precise comment. We have detailed our amendments below.

- Line 54: Brueelia is in Ischnocera, not Amblycera. There has been previous genomic work on Amblycera (e.g., Johnson et al. 2018), but these studies were not full genomic assemblies. Be sure to identify Brueelia as Ischnocera and refer to studies like Johnson et al. (2018) when comparing to Amblycera.

Response: Thanks for your reminder. Corrected accordingly.

- Please provide more information about the cox1 sequencing. Extraction? PCR? And how did you use the cox1 data to confirm species ID? BLAST comparisons?

Response: Thanks for the suggestion. Total genomic DNA were extracted from individual louse using the TIANamp Genomic DNA Kit (Tiangen, Beijing, China) following the manufacturer’s instructions. The COI fragments were amplified by PCR and were sequenced by Sanger sequencing. We also added description in **Methods**. After BLAST comparison, we confirm these COI sequences are from the same species. After receiving this revision, we also assembled mitogenome from the Illumina sequencing library, and this assembled COI sequence is also quite similar to COI fragments by PCR.

- Please clarify what you mean by "genome survey" with the Illumina data. Are you referring to the GenomeScope analysis?

Response: Thanks for this question and sorry for the ambiguity. The genome survey is mean to estimate the genome size, heterozygosity, and duplication of the genome using JELLYFISH version 2.1.3 and GenomeScope version 2.0. We also clarify in **Methods**.

- Why did you use assembly size to choose your assembly? The WTDBG2 and Flye assemblies were very close in size, but the Flye assembly had fewer contigs and a better BUSCO score.

Response: Thanks for this question. Among WTDBG2 and Flye, WTDBG2 produced a larger genome size (155 Mb) and a longer contig N50 (27.42 Mb), therefore the genome assembly from WTDBG2 was used. In the old Revised version, we have stated that: "At the contig level, we generated a final genome assembly of 155 Mb using WTDBG2 as this tool produced the largest contig N50 compared with other tools we tried".

- Line 186 regarding TimeTree seems misplaced? Please edit this sentence.

Response: Corrected accordingly.

- Line 474: What do you mean by "the origin of the individuals was pure?" I think better to rephrase to reinforce your point that your pooled samples are likely genetically very similar and highly inbred (even though not intentionally inbred in a controlled environment).

Response: Thanks for the suggestion. We rephrased this sentence to reinforce our point.

- Do you cox1 data give you any insight about the genetic diversity among your samples? If cox1 is very similar among your samples, this gives you further evidence that your samples are genetically similar.

Response: According to the BLAST comparison, COI fragments are almost the same among our sequenced samples. We think this is due to our louse are collected from several chickens in the same room of the chicken farm.

- Line 479: I do not think *Menopon* is the earliest diverging lineage of Amblycera. Do you mean that Amblycera is sister to the rest of parasitic lice?

Response: Thanks for your reminder. We mean that Amblycera is sister to the rest of parasitic lice. We corrected this sentence.

Again, we thank you and the reviewer for the invaluable comments that have greatly improved our manuscript!

Sincerely yours,

Fan Song

Fan Song, Associate Professor

Department of Entomology

China Agricultural University

Click here to access/download;Figure;Figure 1.pdf

Figure 1

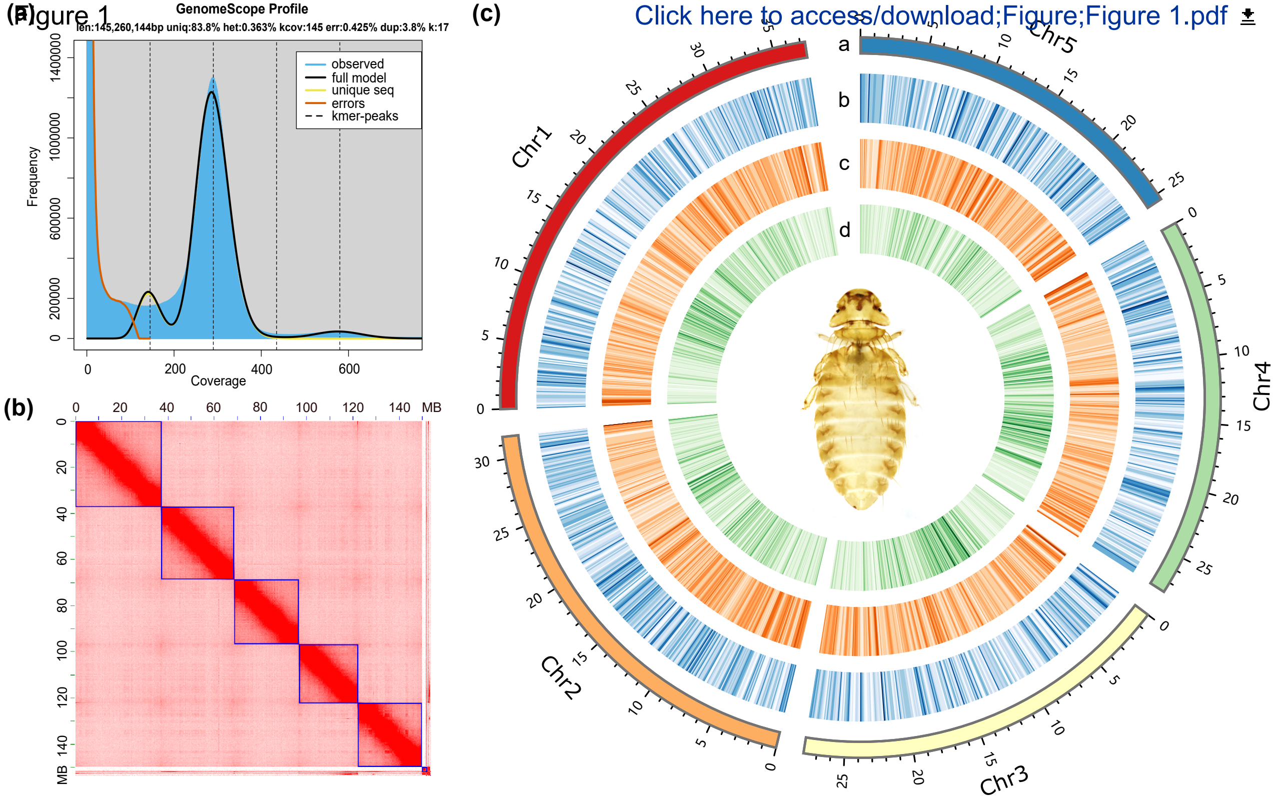

Figure 2

Click here to access/download;Figure;Figure 2.pdf

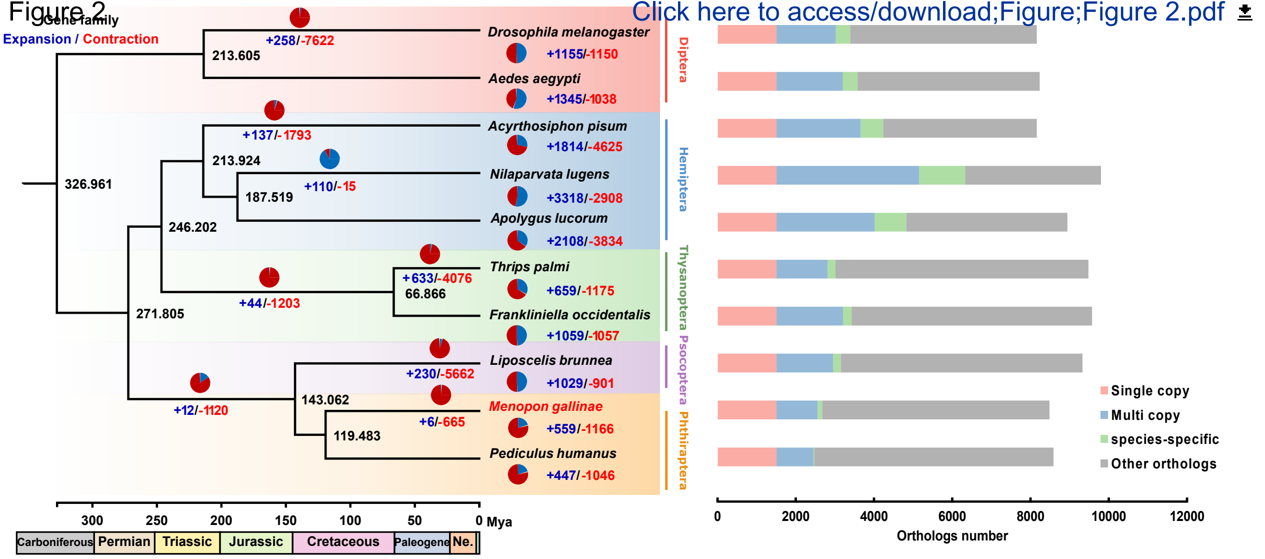

Figure 3

Click here to access/download;Figure;Figure 3.pdf

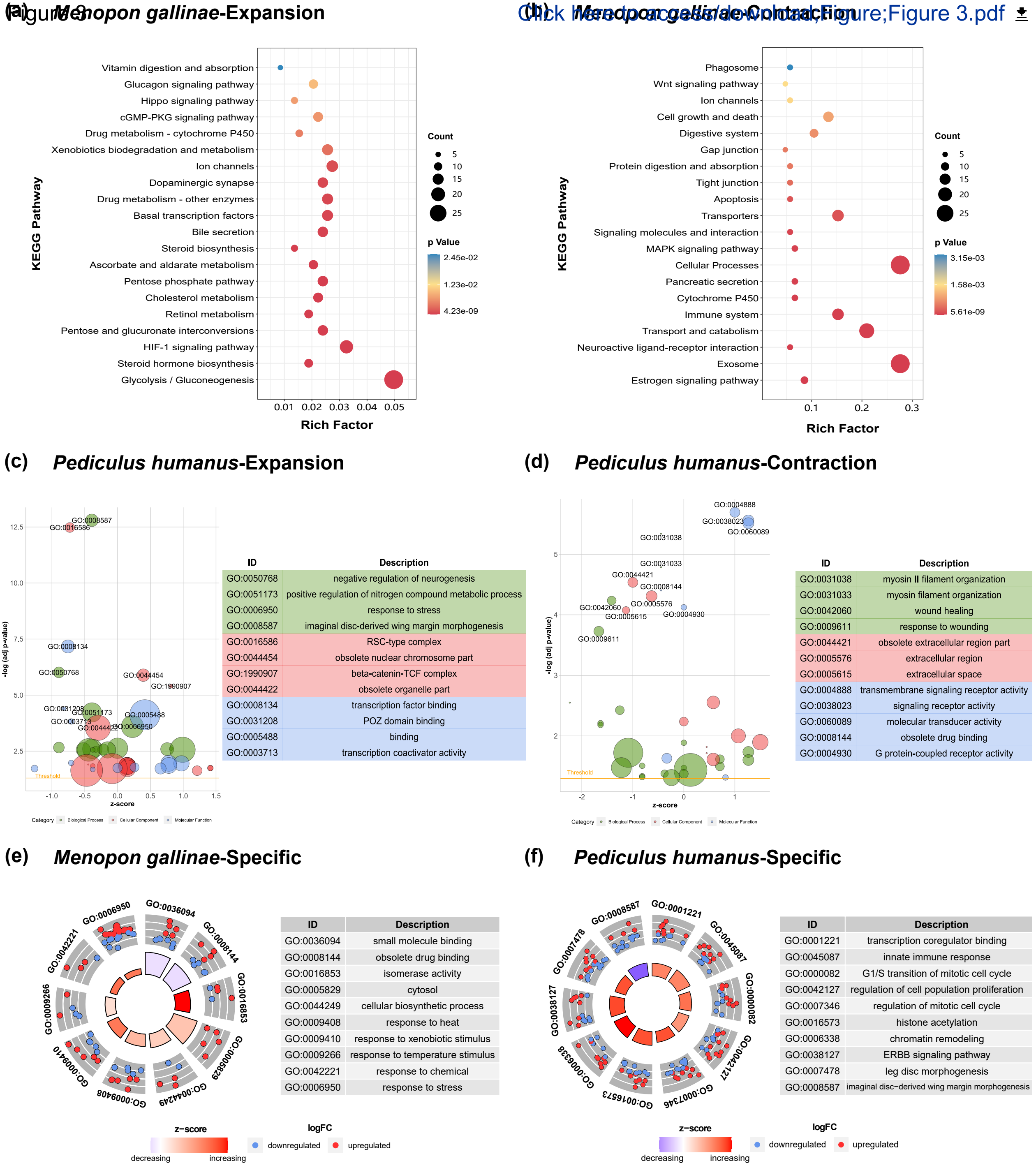

(c) Pediculus humanus-Expansion

ID	Description
GO:0050768	negative regulation of neurogenesis
GO:0051173	positive regulation of nitrogen compound metabolic process
GO:0006950	response to stress
GO:0008587	imaginal disc-derived wing margin morphogenesis
GO:0016586	RSC-type complex
GO:0044454	obsolete nuclear chromosome part
GO:1990907	beta-catenin-TCF complex
GO:0044422	obsolete organelle part
GO:0008134	transcription factor binding
GO:0031208	POZ domain binding
GO:0005488	binding
GO:0003713	transcription coactivator activity

(d) Pediculus humanus-Contraction

ID	Description
GO:0031038	myosin II filament organization
GO:0031033	myosin filament organization
GO:0042060	wound healing
GO:0009611	response to wounding
GO:0044421	obsolete extracellular region part
GO:0005576	extracellular region
GO:0005615	extracellular space
GO:0004888	transmembrane signaling receptor activity
GO:0038023	signaling receptor activity
GO:0060089	molecular transducer activity
GO:0008144	obsolete drug binding
GO:0004930	G protein-coupled receptor activity

(e) Menopon gallinae-Specific

ID	Description
GO:0036094	small molecule binding
GO:0008144	obsolete drug binding
GO:0016853	isomerase activity
GO:0005829	cytosol
GO:0044249	cellular biosynthetic process
GO:0009408	response to heat
GO:0009410	response to xenobiotic stimulus
GO:0009266	response to temperature stimulus
GO:0042221	response to chemical
GO:0006950	response to stress

(f) Pediculus humanus-Specific

ID	Description
GO:0001221	transcription coregulator binding
GO:0045087	innate immune response
GO:0000082	G1/S transition of mitotic cell cycle
GO:0042127	regulation of cell population proliferation
GO:0007346	regulation of mitotic cell cycle
GO:0016573	histone acetylation
GO:0006338	chromatin remodeling
GO:0038127	ERBB signaling pathway
GO:0007478	leg disc morphogenesis
GO:0008587	imaginal disc−derived wing margin morphogenesis

Figure 4

Click here to access/download;Figure;Figure 4.pdf

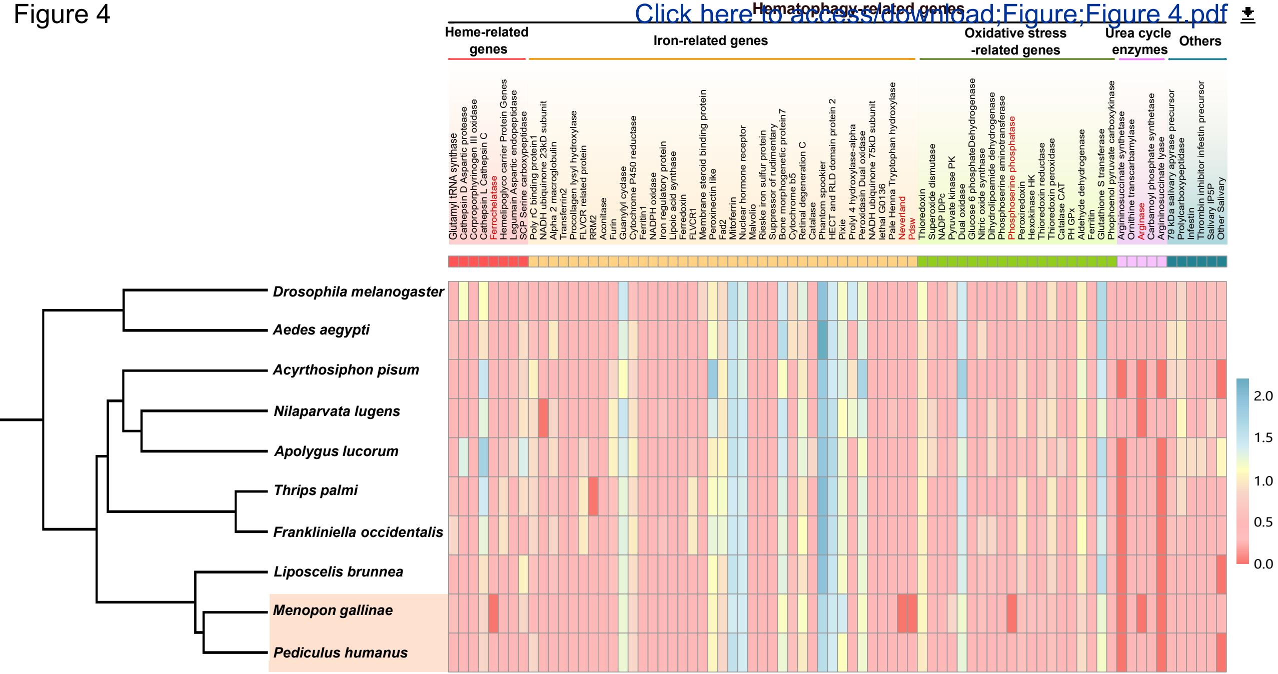

Figure 5

Click here to access/download;Figure;Figure 5.pdf

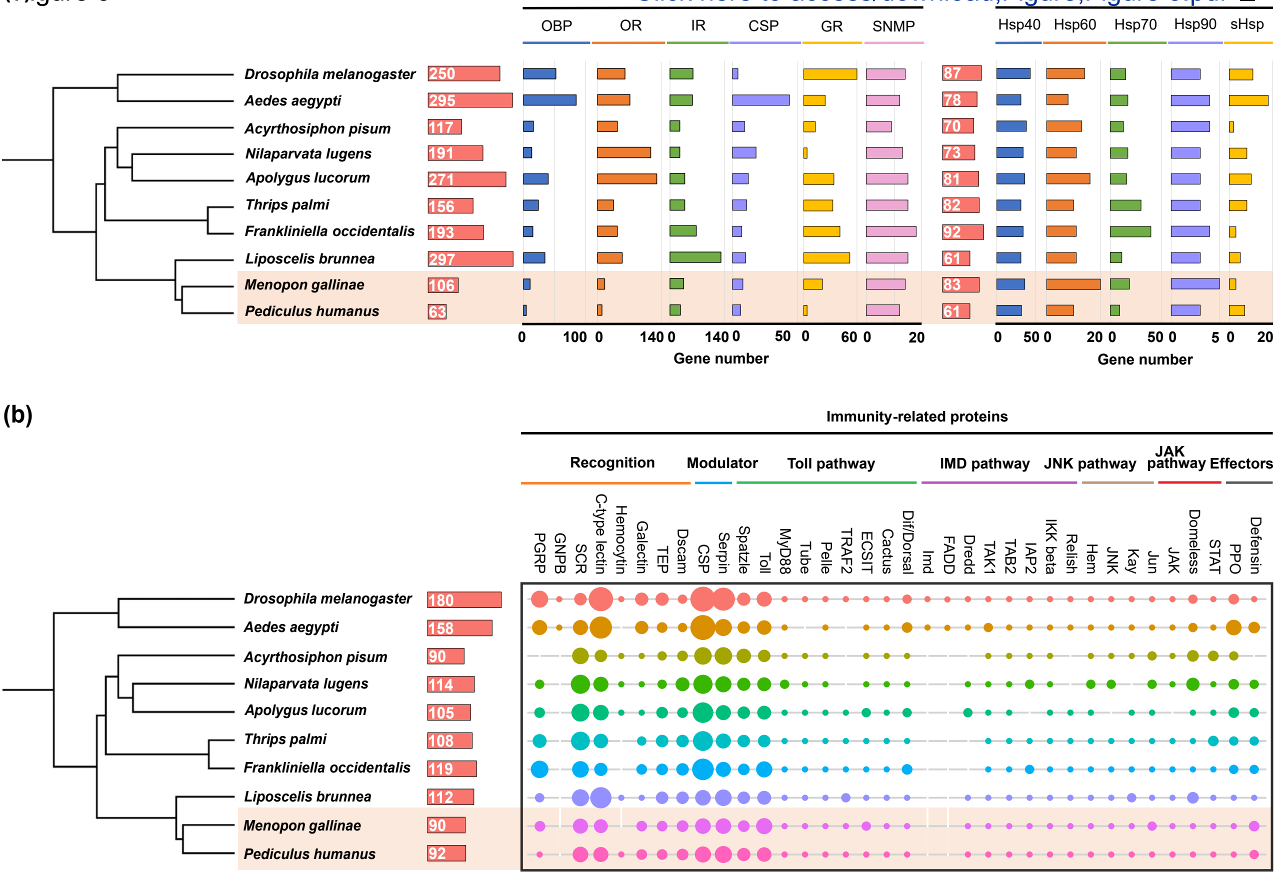

Figure 6

Click here to access/download;Figure;Figure 6.pdf

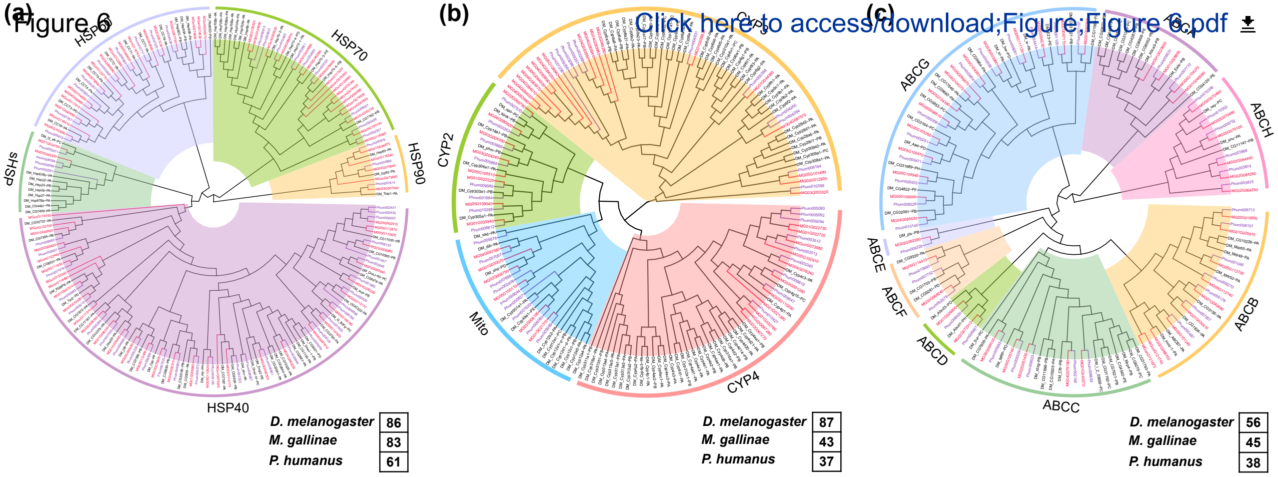

Figure 7

Click here to access/download;Figure;Figure 7.pdf

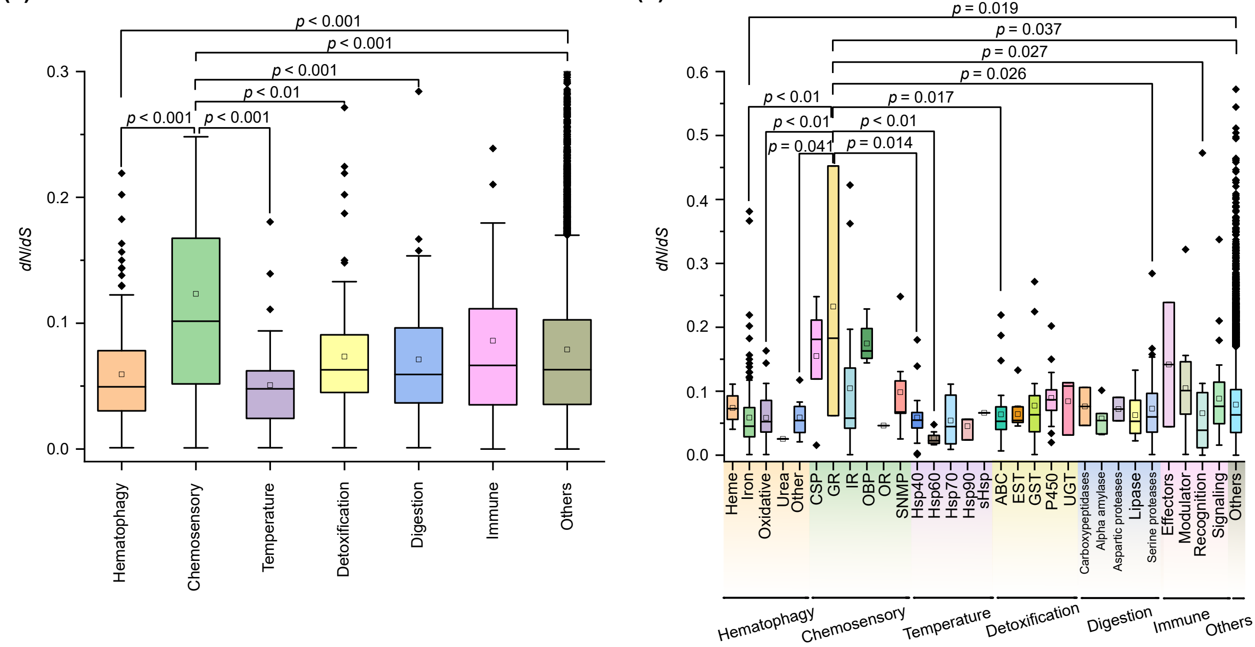

Click here to access/download
Supplementary Material
Xu Ye-Supplemental Information-FigureS1-S3.docx

Click here to access/download

Supplementary Material

Xu Ye-Supplemental Information-TableS1-S22.xlsx
