# Supplementary material for: Chromosome-level genome of the poultry shaft louse Menopon gallinae provides insight into the host-switching and adaptive evolution of parasitic lice
Source: Gigascience. 2024 Feb 19;13:giae004. doi: 10.1093/gigascience/giae004 (PMC10904027; doi:10.1093/gigascience/giae004)
Supplement: giae004_Response_to_Reviewer_Comments_Original_Submission [file giae004_response_to_reviewer_comments_original_submission.pdf]

Author's Response To Reviewer Comments

Dear Editor Hongfang Zhang:

Thank you for handling our article entitled “Chromosome-level genome of the poultry shaft louse Menopon gallinae provides insight into the host-switching and adaptive evolution of parasitic lice”. (ID: GIGA-D-23-00237).
We are grateful to the valuable comments of you and the reviewers. We carefully revised the issue mentioned in the reviewer's comments. Please see our point-to-point response below.

Point-to-point response:

Reviewer reports:

Reviewer #1: Comments:

In this interesting study, the authors report a chromosome-level genome of the poultry louse Menopon gallinae and then use these data to perform a comprehensive comparative genomics analysis to explore the genetic basis of host-switching and adaptive evolution in parasitic lice. They found a number of gene families (such as those related to haematophagy, detoxification and immunity) that may play a key role in host switching in parasitic lice. Here, I have some comments that may be useful in improving the quality of the paper.

Response: We thank the reviewer for the precise comment. We have detailed our amendments below.

Main issues:
A large number of gene loss events are described in the paper, but the authors do not provide a detailed method for identifying gene loss. Therefore, I cannot say whether the gene loss described by the authors is a loss in the OGS gene set or a real loss in the genome. In addition, the authors may also need to conduct pseudogene analysis.

Response: Thanks for the suggestion. We revised to provide a detailed method for identifying gene loss and conducting pseudogene analysis in Methods (Lines 216-221 in Revised_manuscript_changes_tracked.docx). For the putative lost genes in species, we used Exonerate version 2.4.0 to search those protein sequences in other insects against the genome. If the sequence was not mapped to the genome, this might be a gene loss. If the target protein sequence was not mapped to the genome, or aligned to the genome but the genome sequence does not have a complete gene structure (which indicates a pseudogene), then this protein is considered to be lost in this species. In our study, we have described gene loss as genes that were not identified in both the protein and genome sequences. We have also provided a brief explanation of gene loss in the Results section (Lines 394-396, Line 418-419).

This paper deals with the annotation of many multi-gene families. The author simply describes the process of gene annotation as manual annotation, but does not present the specific process. Therefore, the reader cannot tell if the author used some manipulation to try to find genes that were present in the genome sequence but not found by the automated genome annotation.

Response: Thanks for the question. We described the specific process in detail of manual gene annotation in Methods (Lines 210-222). For gene family annotation, we searched these gene families against the protein sequences of 10 species with BLAST version 2.12.0. For the proteins annotated in Pfam database (http://pfam-legacy.xfam.org/), we confirmed the domains of this protein by HMMER version 3.0. For the putative lost genes in species, we used Exonerate version 2.4.0 to search those protein sequences in other insects against the genome. If the sequence was not mapped to the genome or aligned to the genome but not have a complete gene structure, this might be a gene loss. Finally, each candidate gene was manually inspected and divided into subfamilies.

Minor things:
Line 276, why use "re-" here?

Response: Thanks for this question. We previously identified the expanded and contracted gene families in bird lice or mammal lice alone, and here we investigated the expanded and contracted gene families in the ancestor of two parasitic lice. However, to avoid misleading, we deleted “re-”.

Line 320-321, I don't think the use of "similar" is appropriate.

Response: Thanks for the suggestion. We change “a similar amount of … to…” to “more … than…”.

Reviewer #2: Comments:

The authors assembled a chromosome-level genome from pooled individuals of Menopon gallinae. The authors used several genomic sequencing approaches and computational methods to assemble and annotate the genome. They then used a comparative genomics approach to investigate the genomics of host switching from birds to mammals. The resulting assembly and annotation indicated there are five chromosomes in M. gallinae, with gene number that is similar to previously assembled louse genomes. The gene content and signatures of selection varied across different genomes of lice and their close relatives. The functions of some of these genes are consistent with environmental changes associated with switching from bird to mammal hosts.
The genome assembly of an amblyceran louse is a valuable contribution to insect genomics and louse genomics more specifically. There are very few genomes available for lice, despite their importance as a system for understanding host-parasite ecology and evolution. This assembly will be a useful genomic resource.

Response: We thank the approval comments of the reviewer. We have responded to all your concerns below.

However, I have concerns about the manuscript that should be addressed. Most of my comments regard the framework, context, and presentation, but I also have some concerns/questions about methodology.
1) I applaud the authors for going beyond a straight-forward genome assembly/annotation and using their novel genome assembly to address a relevant question in comparative genomics.
However, it is not clear to me how this study is necessary to explore the genomics of bird to mammal host switches. There were likely multiple host switches from birds to mammals, including multiple switches within Amblycera. It isn't necessary to include an amblyceran louse; a genomic comparison between the Columbicola genome (a bird louse) and the human louse genome could suffice. I think the genome of this amblyceran is a valuable contribution, but I think its usefulness in understanding bird-mammal host transitions is overstated. Perhaps future genomic comparisons between bird and mammal lice within Amblycera would be more appropriate for addressing this question.

Response: Thanks for the comments. We acknowledge that our study does not aim to directly address the questions regarding bird to mammal host switches. Our research serves as a valuable reference in this regard. Host-switching is a complex process and we do not claim to address this issue solely based on these two genomes. Instead, our aim was to provide a reference point for understanding host-switching. By comparative genomics analysis of these two insects, we aim to lay the foundation for future research in this field. In response to your concerns, we have made some modifications to our wording to provide a more conservative and suitable representation of our study. For example, we rephrased the last sentence of Abstract (Line 33-34) and Conclusions (Line 549).

2) You do not include references to some relevant work. For example, Sweet et al. (2023) recently published a genome assembly (not chromosome-level, but neither is the human louse genome) from a single individual Brueelia louse. There are also recent studies that include whole genome sequence data from several species amblyceran lice (including Johnson et al. 2018). Although these are not genome assemblies or annotations, these are important genomic data and should be mentioned. At the very least,

the Introduction should clarify that you mean there is a lack of *assembled and annotated* genomes from Amblycera.

Response: Thanks for the suggestion. We added the research of Sweet et al. (2023) (Lines 53-54) and Johnson et al. (2018) (Lines 62-64) as you required to Introduction. We also clarify there is a lack of *assembled and annotated* genomes from Amblycera in Introduction.

3) How did you confirm the species ID for the samples? Also, where did the 1600 samples come from? The same poultry farm?

Response: Thanks for this question. We added described the details in Methods (Lines 94-97). The 1600 individuals were simultaneously collected from several chickens in adjacent cages in the same room on the same chicken farm. Species identification was determined using a combination of morphological identification under the microscope according to Price et al. (2003) and molecular identification through sequencing of COI fragments (~550 bp).

4) Why did you use Illumina for polishing? As I understand it, PacBio does not recommend polishing HiFi reads. It's also unclear how you used the Illumina reads. Please add more justification and details about the Illumina methods.

Response: Thanks for the question. We apologize for the typo. We did not use polishing. We corrected accordingly (Line 105). We also added more justification and details about the Illumina methods (Line 122).

5) Why not use a more established assembly pipeline for HiFi reads, such as HiCanu, HiFiasm, IPA, or Flye?

Response: Following your suggestion, we have added HiCanu, HiFiasm, and Flye software in the Methods section for assembling HiFi reads (Lines 128-136). We also have added the corresponding results to the newly added Table 2 in the Results section (Lines 254-256).

6) It is unclear why you only used single copy orthologs to identify gene families in a phylogenetic context. Wouldn't you want to include potential paralogs when looking at the evolution of gene families?

Response: Thanks for this question. In the old version we have stated that: based on the results of OrthoFinder, gene family clusters were divided into five categories, (1) single-copy genes in all species, (2) multiple-copy genes in at least one species, (3) species-specific genes (genes absent in other N-1 species), and (4) other genes. This means that both single copy and paralogs were used in the downstream analyses. In the revised manuscript, we re-emphasized that OrthoFinder was used to find gene families including single copy genes and paralogous gene families (Lines 172-173).

7) Many of the methods are not clearly justified. For example, why did you estimate divergence times? Please be sure to briefly explain why A) why you did a specific analysis and B) why you chose the specific methods/programs. Currently, the Methods sections reads more like a list of analyses, rather than a narrative that justifies methodological choices.

Response: Thanks for the suggestion. For estimate divergence times, we make the following explanation. A) In brief, divergence time was required for the expansion and contraction analyses. We also provide a detailed method for estimate divergence times in Methods (Lines 198-200). B) MCMCTree was used to estimate divergence time according to your suggestion in the later questions in Methods (Lines 184-186). We added a briefly explain before most of the analysis in Methods sections.

8) Why did you use RAxML for one tree estimation but IQ-Tree for another phylogenetic analysis?

Response: Thanks for this question. In the revised manuscript, we consistently used IQ-Tree to construct the phylogenetic trees in Methods (Lines 180-184). We also updated Figure 2.

9) Could the heterozygosity rate be misleading? Previous louse genome assemblies from pooled samples

(Columbicola and Pediculus) were from inbred samples, so this was not as much of an issue. Do you have any sense for the genetic diversity on a host individual (i.e., infrapopulation)? On the same host at the same location?

Response: Thanks for the comments. We added heterozygosity to Table 1. Although our Menopon gallinae individuals are not from inbred population, they were simultaneously collected from several chickens in adjacent cages in the same room on the same chicken farm. Since the chicken farm was established, the chickens in the farm have never been in contact with the chickens outside. Therefore, the lice on the hosts are likely to have a highly homogeneous genetic background. Accordingly, the heterozygosity in our samples was as low as 0.363%, the level of which was lower than that of feather louse B. nebuosa (1.2%) (only one individual was used), melon thrips Thrips palmi (1.32%) and mirid predator Cyrtorhinus lividipennis (1.7%). We also added explain in Discussion (Lines 464-470).

10) It's unclear why you compared to Liposcelis in the Results. You do not introduce this published genome earlier, and you do not compare to the other published louse genomes in the Results. I recommend introducing Liposcelis more explicitly in the Introduction.

Response: Thanks for the suggestion. By comparing Liposcelis with the common ancestor of mammal lice and bird lice, we would better understand the genetic difference between non-parasitic lice and parasitic lice. We added description of Liposcelis to the Introduction (Lines 64-67). We also compared our new genome to the other published louse genomes in the Results (Lines 259-272).

11) The dN/dS ratios seem pretty low, which to me suggests strong purifying selection. I think this is worth developing in the Discussion. Also, could there be issues with calculating dN/dS ratios from pooled samples, especially in this case where the Menopon samples were not intentionally inbred?

Response: Thanks for your reminder. We added the discussion of dN/dS ratios to Discussion (Lines 470-474). It's worth noting that the low dN/dS ratios in both important gene families of M. gallinae and P. humanus indicate that they have undergone strong purifying selection, meaning that the majority of deleterious mutations have been eliminated. Although not inbred population of M. gallinae were used to conduct genome sequencing and assembly, the origin of the individuals was pure.

12) The Discussion seems a little underdeveloped. I would expect more comparisons between other louse genomes. There is some comparison, but I think more details (e.g., chromosome number, etc.) would strengthen the manuscript.

Response: Thanks for the suggestion. We added more comparisons between other louse genomes in Discussion (Lines 474-483), including the comparison of chromosome numbers (Lines 479-483).

Minor:
- Line 43 should be a new paragraph.

Response: Thanks. Corrected accordingly.

- Line 112 is redundant.

Response: Corrected accordingly.

- Line 124: what ortholog set did you use for BUSCO?

Response: Thanks for your reminder. We used ortholog set is insecta_odb10 for BUSCO. We have added this description to the revised manuscript.

- Line 165: Why use r8s instead of a more commonly used method such as MCMCTree? Regardless, please provide more details about how you estimated ages. What parameters did you use? Where did the TIMETREE dates come from?

Response: Thanks for this question. In the revised manuscript, we updated the results using the more commonly used method MCMCTree. We updated Figure 2. Accordingly, CAFE software was used to re-analyze the expanded and contracted gene families. And we re-analyze gene families GO and KEGG enrichment. We updated Figure 2, Figure 3 and Table S5-S14. The detailed processes of age estimation, parameters, and sources were added to the Methods section (Lines 184-186).

- Line 256: It's unclear where the 137,947 genes come from. Is this summed across all species?

Response: Thanks for this question and sorry for the ambiguity. Yes, the 137,947 genes are the total number of genes identified in orthogroups of all ten insect species in this study. We clarified this issue in Results (Lines 315-316).

- Line 395: But see Sweet et al. (2023).

Response: Thanks for your reminder and we apologize for the misleading expression. Sweet et al presented the first genome of Amblycera, Brueelia nebuosa. In our work, we present the first chromosome-level genome of Amblycera, Menopon gallinae. We clarified this issue in revised manuscript.

- Line 402: I don't find the levels of heterozygosity particularly low, although those values are lower than in Brueelia, Columbicola, or Pediculus. Could you provide comparisons from other insects? The GenomeScope plot also shows some evidence of two peaks, which suggests a heterozygous genome.

Response: This is a good question. We added more heterozygosity comparisons from other insects in Discussion. The heterozygosity in our samples was as low as 0.363%, the level of which was lower than that of feather louse B. nebuosa (1.2%) (only one individual was used), melon thrips Thrips palmi (1.32%) and mirid predator Cyrtorhinus lividipennis (1.7%) (Lines 467-470).
In the GenomeScope plot results, the two peaks represent different aspects of the genome. The first peak graph represents heterozygous peak and typically corresponds to heterozygosity within the genome. The second peak graph represents homozygous peak and typically corresponds to the haploid genome or the main genomic component. If the height of the second (homozygous) peak is much higher than the first (heterozygous) peak, it suggests a genome with low heterozygosity. In our Menopon gallinae genome, the GenomeScope plot shows that the second peak (homozygous, genome unique length: 121,570,805 bp) is much higher than the first peak (heterozygous, genome repeat length: 23,503,398 bp), suggesting low heterozygosity in the M. gallinae genome.

- I'm curious why the acknowledgments section is so brief, without any mention of the poultry farm or any other resources (computational, etc.) that were involved in the project.

Response: Thanks for your reminder. In the new version, we acknowledged the poultry farm and other relevant peoples.

- Table 1 should also include comparisons with Columbicola, Brueelia, and Liposcelis

Response: Thanks for the suggestion. We have revised the original Table 1.

Again, we thank you and the reviewers for the invaluable comments that have greatly improved our manuscript!

Sincerely yours,
Fan Song, Associate Professor

Department of Entomology
China Agricultural University
No.2 Yuanmingyuan West Road
Beijing 100193, China
