# Supplementary material for: Chromosome-level genome of the poultry shaft louse Menopon gallinae provides insight into the host-switching and adaptive evolution of parasitic lice
Source: Gigascience. 2024 Feb 19;13:giae004. doi: 10.1093/gigascience/giae004 (PMC10904027; doi:10.1093/gigascience/giae004)
Supplement: giae004_Response_to_Reviewer_Comments_Revision_1 — Xinhai Ye -- 9/4/2023 [file giae004_response_to_reviewer_comments_revision_1.pdf]

Author's Response To Reviewer Comments

Dear Editor Hongfang Zhang:
Thank you for handling our article entitled "Chromosome-level genome of the poultry shaft louse Menopon gallinae provides insight into the host-switching and adaptive evolution of parasitic lice". (ID: GIGA-D-23-00237R1).
We are grateful to the positive comments of you and the reviewer. We carefully revised the issue mentioned in the reviewer's comments. Please see our point-to-point response below.

Point-to-point response:
Reviewer #2: Comments:

Thank you for your thorough response to my comments! I think you improved the manuscript considerably. However, I think there are a few more comments that should be addressed:

Response: We thank the reviewer for the precise comment. We have detailed our amendments below.

- Line 54: Brueelia is in Ischnocera, not Amblycera. There has been previous genomic work on Amblycera (e.g., Johnson et al. 2018), but these studies were not full genomic assemblies. Be sure to identify Brueelia as Ischnocera and refer to studies like Johnson et al. (2018) when comparing to Amblycera.

Response: Thanks for your reminder. Corrected accordingly.

- Please provide more information about the cox1 sequencing. Extraction? PCR? And how did you use the cox1 data to confirm species ID? BLAST comparisons?

Response: Thanks for the suggestion. Total genomic DNA were extracted from individual louse using the TIANamp Genomic DNA Kit (Tiangen, Beijing, China) following the manufacturer's instructions. The COI fragments were amplified by PCR and were sequenced by Sanger sequencing. We also added description in Methods. After BLAST comparison, we confirm these COI sequences are from the same species. After receiving this revision, we also assembled mitogenome from the Illumina sequencing library, and this assembled COI sequence is also quite similar to COI fragments by PCR.

- Please clarify what you mean by "genome survey" with the Illumina data. Are you referring to the GenomeScope analysis?

Response: Thanks for this question and sorry for the ambiguity. The genome survey is mean to estimate the genome size, heterozygosity, and duplication of the genome using JELLYFISH version 2.1.3 and GenomeScope version 2.0. We also clarify in Methods.

- Why did you use assembly size to choose your assembly? The WTDBG2 and Flye assemblies were very close in size, but the Flye assembly had fewer contigs and a better BUSCO score.

Response: Thanks for this question. Among WTDBG2 and Flye, WTDBG2 produced a larger genome size (155 Mb) and a longer contig N50 (27.42 Mb), therefore the genome assembly from WTDBG2 was used. In the old Revised version, we have stated that: "At the contig level, we generated a final genome assembly of 155 Mb using WTDBG2 as this tool produced the largest contig N50 compared with other tools we tried".

- Line 186 regarding TimeTree seems misplaced? Please edit this sentence.

Response: Corrected accordingly.

- Line 474: What do you mean by "the origin of the individuals was pure?" I think better to rephrase to reinforce your point that your pooled samples are likely genetically very similar and highly inbred (even though not intentionally inbred in a controlled environment).

Response: Thanks for the suggestion. We rephrased this sentence to reinforce our point.

- Do you cox1 data give you any insight about the genetic diversity among your samples? If cox1 is very similar among your samples, this gives you further evidence that your samples are genetically similar.

Response: According to the BLAST comparison, COI fragments are almost the same among our sequenced samples. We think this is due to our louse are collected from several chickens in the same room of the chicken farm.

- Line 479: I do not think Menopon is the earliest diverging lineage of Amblycera. Do you mean that Amblycera is sister to the rest of parasitic lice?

Response: Thanks for your reminder. We mean that Amblycera is sister to the rest of parasitic lice. We corrected this sentence.

Again, we thank you and the reviewer for the invaluable comments that have greatly improved our manuscript!

Sincerely yours,

Fan Song, Associate Professor
Department of Entomology
China Agricultural University
