# Supplementary material for: Chromosome-level genome of the poultry shaft louse Menopon gallinae provides insight into the host-switching and adaptive evolution of parasitic lice
Source: Gigascience. 2024 Feb 19;13:giae004. doi: 10.1093/gigascience/giae004 (PMC10904027; doi:10.1093/gigascience/giae004)
Supplement: giae004_Reviewer_2_Report_Original_Submission — Drew Sweet -- 9/21/2023 [file giae004_reviewer_2_report_original_submission.pdf]

Reviewer Report

Title: Chromosome-level genome of the poultry shaft louse Menopon gallinae provides insight into the host-switching and adaptive evolution of parasitic lice

Version: Original Submission **Date:** 9/21/2023

Reviewer name: Drew Sweet

Reviewer Comments to Author:

The authors assembled a chromosome-level genome from pooled individuals of Menopon gallinae. The authors used several genomic sequencing approaches and computational methods to assemble and annotate the genome. They then used a comparative genomics approach to investigate the genomics of host switching from birds to mammals. The resulting assembly and annotation indicated there are five chromosomes in M. gallinae, with gene number that is similar to previously assembled louse genomes. The gene content and signatures of selection varied across different genomes of lice and their close relatives. The functions of some of these genes are consistent with environmental changes associated with switching from bird to mammal hosts.

The genome assembly of an amblyceran louse is a valuable contribution to insect genomics and louse genomics more specifically. There are very few genomes available for lice, despite their importance as a system for understanding host-parasite ecology and evolution. This assembly will be a useful genomic resource.

However, I have concerns about the manuscript that should be addressed. Most of my comments regard the framework, context, and presentation, but I also have some concerns/questions about methodology.

1) I applaud the authors for going beyond a straight-forward genome assembly/annotation and using their novel genome assembly to address a relevant question in comparative genomics. However, it is not clear to me how this study is necessary to explore the genomics of bird to mammal host switches. There were likely multiple host switches from birds to mammals, including multiple switches within Amblycera. It isn't necessary to include an amblyceran louse; a genomic comparion between the Columbicola genome (a bird louse) and the human louse genome could suffice. I think the genome of this amblyceran is a valuable contribution, but I think its usefulness in understanding bird-mammal host transitions is overstated. Perhaps future genomic comparisons between bird and mammal lice within Amblycera would be more appropriate for addressing this question.

2) You do not include references to some relevant work. For example, Sweet et al. (2023) recently published a genome assembly (not chromosome-level, but neither is the human louse genome) from a single individual Brueelia louse. There are also recent studies that include whole genome sequence data from several species amblyceran lice (including Johnson et al. 2018). Although these are not genome assemblies or annotations, these are important genomic data and should be mentioned. At the very least, the Introduction should clarify that you mean there is a lack of *assembled and annotated* genomes from Amblycera.

3) How did you confirm the species ID for the samples? Also, where did the 1600 samples come from? The same poultry farm?
4) Why did you use Illumina for polishing? As I understand it, PacBio does not recommend polishing HiFi reads. It's also unclear how you used the Illumina reads. Please add more justification and details about the Illumina methods.
5) Why not use a more established assembly pipeline for HiFi reads, such as HiCanu, HiFiasm, IPA, or Flye?
6) It is unclear why you only used single copy orthologs to identify gene families in a phylogenetic context. Wouldn't you want to include potential paralogs when looking at the evolution of gene families?
7) Many of the methods are not clearly justified. For example, why did you estimate divergence times? Please be sure to briefly explain why A) why you did a specific analysis and B) why you chose the specific methods/programs. Currently, the Methods sections reads more like a list of analyses, rather than a narrative that justifies methodological choices.
8) Why did you use RAxML for one tree estimation but IQ-Tree for another phylogenetic analysis?
9) Could the heterozygosity rate be misleading? Previous louse genome assemblies from pooled samples (Columbicola and Pediculus) were from inbred samples, so this was not as much of an issue. Do you have any sense for the genetic diversity on a host individual (i.e., infrapopulation)? On the same host at the same location?
10) It's unclear why you compared to Liposcelis in the Results. You do not introduce this published genome earlier, and you do not compare to the other published louse genomes in the Results. I recommend introducing Liposcelis more explicitly in the Introduction.
11) The dN/dS ratios seem pretty low, which to me suggests strong purifying selection. I think this is worth developing in the Discussion. Also, could there be issues with calculating dN/dS ratios from pooled samples, especially in this case where the Menopon samples were not intentionally inbred?
12) The Discussion seems a little underdeveloped. I would expect more comparisons between other louse genomes. There is some comparison, but I think more details (e.g., chromosome number, etc.) would strengthen the manuscript.
Minor:
- Line 43 should be a new paragraph.
- Line 112 is redundant.
- Line 124: what ortholog set did you use for BUSCO?
- Line 165: Why use r8s instead of a more commonly used method such as MCMCTree? Regardless, please provide more details about how you estimated ages. What parameters did you use? Where did the TIMETREE dates come from?
- Line 256: It's unclear where the 137,947 genes come from. Is this summed across all species?
- Line 395: But see Sweet et al. (2023).
- Line 402: I don't find the levels of heterozygosity particularly low, although those values are lower than in Brueelia, Columbicola, or Pediculus. Could you provide comparisons from other insects? The GenomeScope plot also shows some evidence of two peaks, which suggests a heterozygous genome.
- I'm curious why the acknowledgments section is so brief, without any mention of the poultry farm or any other resources (computational, etc.) that were involved in the project.

- Table 1 should also include comparisons with Columbicola, Brueelia, and Liposcelis

Methods

Are the methods appropriate to the aims of the study, are they well described, and are necessary controls included? Choose an item.

Conclusions

Are the conclusions adequately supported by the data shown? Choose an item.

Reporting Standards

Does the manuscript adhere to the journal's guidelines on minimum standards of reporting? Choose an item.

Choose an item.

Statistics

Are you able to assess all statistics in the manuscript, including the appropriateness of statistical tests used? Choose an item.

Quality of Written English

Please indicate the quality of language in the manuscript: Choose an item.

Declaration of Competing Interests

Please complete a declaration of competing interests, considering the following questions:

- Have you in the past five years received reimbursements, fees, funding, or salary from an organisation that may in any way gain or lose financially from the publication of this manuscript, either now or in the future?
- Do you hold any stocks or shares in an organisation that may in any way gain or lose financially from the publication of this manuscript, either now or in the future?
- Do you hold or are you currently applying for any patents relating to the content of the manuscript?
- Have you received reimbursements, fees, funding, or salary from an organization that holds or has applied for patents relating to the content of the manuscript?
- Do you have any other financial competing interests?
- Do you have any non-financial competing interests in relation to this paper?

If you can answer no to all of the above, write 'I declare that I have no competing interests' below. If your reply is yes to any, please give details below.

I declare that I have no competing interests

I agree to the open peer review policy of the journal. I understand that my name will be included on my report to the authors and, if the manuscript is accepted for publication, my named report including any attachments I upload will be posted on the website along with the authors' responses. I agree for my report to be made available under an Open Access Creative Commons CC-BY license (http://creativecommons.org/licenses/by/4.0/). I understand that any comments which I do not wish to be included in my named report can be included as confidential comments to the editors, which will not be published.

Choose an item.

To further support our reviewers, we have joined with Publons, where you can gain additional credit to further highlight your hard work (see: https://publons.com/journal/530/gigascience). On publication of this paper, your review will be automatically added to Publons, you can then choose whether or not to claim your Publons credit. I understand this statement.

Yes Choose an item.
