# Supplementary material for: Chromosome-level genome of the poultry shaft louse Menopon gallinae provides insight into the host-switching and adaptive evolution of parasitic lice
Source: Gigascience. 2024 Feb 19;13:giae004. doi: 10.1093/gigascience/giae004 (PMC10904027; doi:10.1093/gigascience/giae004)
Supplement: giae004_Reviewer_2_Report_Revision_1 — Drew Sweet -- 11/28/2023 [file giae004_reviewer_2_report_revision_1.pdf]

Reviewer Report

Title: Chromosome-level genome of the poultry shaft louse Menopon gallinae provides insight into the host-switching and adaptive evolution of parasitic lice

Version: Revision 1 **Date:** 11/28/2023

Reviewer name: Drew Sweet

Reviewer Comments to Author:

Thank you for your thorough response to my comments! I think you improved the manuscript considerably. However, I think there are a few more comments that should be addressed:
- Line 54: Brueelia is in Ischnocera, not Amblycera. There has been previous genomic work on Amblycera (e.g., Johnson et al. 2018), but these studies were not full genomic assemblies. Be sure to identify Brueelia as Ischnocera and refer to studies like Johnson et al. (2018) when comparing to Amblycera.
- Please provide more information about the cox1 sequencing. Extraction? PCR? And how did you use the cox1 data to confirm species ID? BLAST comparisons?
- Please clarify what you mean by "genome survey" with the Illumina data. Are you referring to the GenomeScope analysis?
- Why did you use assembly size to choose your assembly? The WTDBG2 and Flye assemblies were very close in size, but the Flye assembly had fewer contigs and a better BUSCO score.
- Line 186 regarding TimeTree seems misplaced? Please edit this sentence.
- Line 474: What do you mean by "the origin of the individuals was pure?" I think better to rephrase to reinforce your point that your pooled samples are likely genetically very similar and highly inbred (even though not intentionally inbred in a controlled environment).
- Do you cox1 data give you any insight about the genetic diversity among your samples? If cox1 is very similar among your samples, this gives you further evidence that your samples are genetically similar.
- Line 479: I do not think Menopon is the earliest diverging lineage of Amblycera. Do you mean that Amblycera is sister to the rest of parasitic lice?

Methods

Are the methods appropriate to the aims of the study, are they well described, and are necessary controls included? Choose an item.

Conclusions

Are the conclusions adequately supported by the data shown? Choose an item.

Reporting Standards

Does the manuscript adhere to the journal’s guidelines on [minimum standards of reporting?]() Choose an item.

Choose an item.

Statistics

Are you able to assess all statistics in the manuscript, including the appropriateness of statistical tests used? Choose an item.

Quality of Written English

Please indicate the quality of language in the manuscript: Choose an item.

Declaration of Competing Interests

Please complete a declaration of competing interests, considering the following questions:

- Have you in the past five years received reimbursements, fees, funding, or salary from an organisation that may in any way gain or lose financially from the publication of this manuscript, either now or in the future?
- Do you hold any stocks or shares in an organisation that may in any way gain or lose financially from the publication of this manuscript, either now or in the future?
- Do you hold or are you currently applying for any patents relating to the content of the manuscript?
- Have you received reimbursements, fees, funding, or salary from an organization that holds or has applied for patents relating to the content of the manuscript?
- Do you have any other financial competing interests?
- Do you have any non-financial competing interests in relation to this paper?

If you can answer no to all of the above, write 'I declare that I have no competing interests' below. If your reply is yes to any, please give details below.

I declare that I have no competing interests.

I agree to the open peer review policy of the journal. I understand that my name will be included on my report to the authors and, if the manuscript is accepted for publication, my named report including any attachments I upload will be posted on the website along with the authors' responses. I agree for my report to be made available under an Open Access Creative Commons CC-BY license (http://creativecommons.org/licenses/by/4.0/). I understand that any comments which I do not wish to be included in my named report can be included as confidential comments to the editors, which will not be published.

Choose an item.

To further support our reviewers, we have joined with Publons, where you can gain additional credit to further highlight your hard work (see: https://publons.com/journal/530/gigascience). On publication of this paper, your review will be automatically added to Publons, you can then choose whether or not to claim your Publons credit. I understand this statement.

Yes Choose an item.
